# Redox-Responsive Nanocarrier for Controlled Release of Drugs in Inflammatory Skin Diseases

**DOI:** 10.3390/pharmaceutics13010037

**Published:** 2020-12-29

**Authors:** Keerthana Rajes, Karolina A. Walker, Sabrina Hadam, Fatemeh Zabihi, Fiorenza Rancan, Annika Vogt, Rainer Haag

**Affiliations:** 1 Institute of Chemistry and Biochemistry, Freie Universität Berlin, Takustr. 3, 14195 Berlin, Germany; keerthana.rajes@fu-berlin.de; 2 Clinical Research Center for Hair and Skin Science, Department of Dermatology and Allergy, Charité-Universitaetsmedizin Berlin, Corporate Member of Freie Universität Berlin, Humboldt Universität zu Berlin, and Berlin Institute of Health, 10117 Berlin, Germany; sabrina.hadam@charite.de (S.H.); fatemezabihi@zedat.fu-berlin.de (F.Z.); fiorenza.rancan@charite.de (F.R.); annika.vogt@charite.de (A.V.)

**Keywords:** CMS nanocarriers, disulfide, redox, stimuli responsive, cyclic voltammetry, skin penetration, rapamycin, dexamethasone, anti-inflammatory drugs

## Abstract

A synthetic route for redox-sensitive and non-sensitive core multi-shell (CMS) carriers with sizes below 20 nm and narrow molecular weight distributions was established. Cyclic voltammetric measurements were conducted characterizing the redox potentials of reduction-sensitive CMS while showcasing its reducibility through glutathione and tris(2-carboxyethyl)-phosphine as a proof of concept. Measurements of reduction-initiated release of the model dye Nile red by time-dependent fluorescence spectroscopy showed a pronounced release for the redox-sensitive CMS nanocarrier (up to 90% within 24 h) while the non-sensitive nanocarriers showed no release in PBS. Penetration experiments using ex vivo human skin showed that the redox-sensitive CMS nanocarrier could deliver higher percentages of the loaded macrocyclic dye meso-tetra (*m*-hydroxyphenyl) porphyrin (mTHPP) to the skin as compared to the non-sensitive CMS nanocarrier. Encapsulation experiments showed that these CMS nanocarriers can encapsulate dyes or drugs with different molecular weights and hydrophobicity. A drug content of 1 to 6 wt% was achieved for the anti-inflammatory drugs dexamethasone and rapamycin as well as fluorescent dyes such as Nile red and porphyrins. These results show that redox-initiated drug release is a promising strategy to improve the topical drug delivery of macrolide drugs.

## 1. Introduction

Immunosuppressive drugs are broadly used to treat immune-mediated and autoimmune diseases [1]. Such drugs are also widely used in dermatology for the systemic as well as topical treatment of inflammatory skin diseases. Among macrolide immunosuppressants, rapamycin (also known as sirolimus) is an immunosuppressor that functions through inhibiting T cell proliferation and activation and is an alternative for previously used anti-inflammatory drugs in inflamed skin [2]. However, low skin permeability and high molecular weights limit its dermal accessibility.

Various carrier systems have been developed for drug delivery in the past as they harbor various advantages: they increase the drug solubility through encapsulation, their synthesis is less costly and time consuming than improving the drugs themselves, they decrease the required drug doses, as well as side effects, and can also protect drugs from degradation [3].

Skin harbors various redox-active compounds such as thiols, disulfides, enzymes and reactive oxygen species that help maintaining a redox balance [4]. The reductive and oxidative environment of the skin varies within the different skin layers, which is also known as its redox gradient. The skin barrier, i.e., the stratum corneum (SC), is rich in thiol groups [5]. Its representative redox-active moieties are glutathione (GSH) and reactive oxygen species such as hydrogen peroxide.

Tackling the reductive environment in skin, disulfide-incorporated polymer systems have already been investigated for drug delivery systems (DDSs), resulting in mostly polymeric micelles [6,7,8,9,10,11,12]. Micellar structures are based on self-assembly and their stability is therefore limited by the critical micelle concentration (CMC). Once the concentration becomes lower than the CMC, the micelles can disintegrate and release their cargo, thus leading to unspecific drug delivery despite aiming a specific target site. This can be avoided by using core multi-shell (CMS) structures. In contrast to micelles, CMS nanocarriers are made of a dendritic core molecule, which is covalently attached to various amphiphilic linear molecules that form a double shell structure around it with chemically defined hydrophilic or hydrophobic regions. Thus, they do not rely on the weak intermolecular interactions involved in self-assembled micelles. CMS carriers have broadly modifiable structures that allow the encapsulation of a wide range of hydrophobic and hydrophilic drugs due to their amphiphilic nature [13]. The modification ranges from changes in internal cavities to size increment, leaving a lot of room for drug-specific fine-tuning and target-specific controlled drug release [14].

Targeting the redox-sensitive moieties, a redox-sensitive CMS (rsCMS) nanocarrier, capable of releasing encapsulated drugs at highly reductive sites in a triggered fashion, was designed. A disulfide unit was implemented into the hydrophobic inner shell of the nanocarrier as disulfide has been reported to be an efficient functional group for reduction-sensitive drug delivery systems [15,16,17]. This work focuses on the synthesis and characterization of the CMS carriers and their redox responsiveness. Therefore, their redox potentials were measured to compare with common reducing agents. Controlled in vitro drug release experiments were performed. Tris(2-carboxyethyl)-phosphine (TCEP) and GSH were chosen as reducing agents to probe the reductive behavior of the rsCMS nanocarrier. TCEP is a commonly used reducing agent known for its moderate reducing properties. GSH is known to cleave disulfide bonds by attaching to one cleaved thiol, forming a disulfide bond, liberating the other half of the disulfide as a thiol, thus being a slight reducing agent in comparison to TCEP. It is hypothesized that the rsCMS nanocarrier can release encapsulated drugs upon reduction in a controlled fashion at the site of inflammation. To investigate its efficacy, a comparative non-redox sensitive nanocarrier (ccCMS) omitting the disulfide moiety was synthesized.

Skin penetration experiments were conducted as a proof of concept. Finally, the drug loading capacity was also tested. The chosen hydrophobic drugs rapamycin and dexamethasone are anti-inflammatory agents. Previous studies with CMS systems show for hydrophobic drugs high drug densities in the inner shell or between the hydrophobic inner shell building blocks [3]. Locating the redox-sensitive moieties at the middle of the inner shell close to the encapsulated drug will allow a direct effect of the reductive environment on the encapsulated drugs.

## 2. Materials and Methods

### 2.1. Chemical Reagents

mPEG-OH, anhydrous CH_2_Cl_2_, docosanedioic acid **6b**, KI, dexamethasone and streptomycin were purchased from Sigma-Aldrich (Merck KGaA, Darmstadt, Germany). Triethyl amine and anhydrous DMSO were purchased from Acros Organics. Mesyl chloride, I_2_, 11-mercaptoundecanoic acid **5a** and *N*-hydroxysuccinimide (NHS) were purchased from Aldrich. NH_3_ 25% solution, NaSO_4_, MeOH, Na_2_SO_3_ and 1-ethyl-3-(3-dimethylaminopropyl) carbodiimide (EDCI) were purchased from Carl Roth (Carl Roth GmbH + Co. KG, Karlsruhe, Germany). NaOH and HCl were bought from Fisher Scientific (Fisher Scientific GmbH, Schwerte, Germany). 2-Morpholinoethanesulfonic acid (MES), GSH, oxidized glutathione (GSSG), TCEP and fluorescein isothiocyanate (FITC) were purchased from TCI (TCI Deutschland GmbH, Eschborn, Germany). Hydroxyethyl cellulose (HEC) gel was purchased from Ashland (Düsseldorf, Germany). Diethyl ether, acetone and acetonitrile were purchased from VWR Chemicals (Avantor, Darmstadt, Germany). Meso-tetra (*m*-hydroxyphenyl) porphyrin (mTHPP) was purchased from Biolitec (Jena, Germany). Pheophorbide A (PhA) was purchased from Frontier scientific (Logan, UT, USA). Rapamycin was bought from LC Laboratories (Woburn, MA, USA). Aminated hyperbranched polyglycerol (hPG-NH_2_, 10 kDa, 70% functionalized, 20 mg/mL in MeOH) was synthesized according to Roller et al. [18].

### 2.2. Instrumentation and Methods

#### 2.2.1. Synthesis of the Starting Materials

*mPEG-OMs **3***

Based on a procedure [19], methoxy poly (ethylene glycol) (mPEG-OH) **2** (10.04 g, 13.4 mmol, 1.0 equiv.) was dissolved in anhydrous CH_2_Cl_2_ (13.5 mL, 1 mL/mmol) and cooled with an ice bath. Triethylamine (4.5 mL, 33.1 mmol, 2.5 equiv.) was added dropwise after adding mesyl chloride (3.0 mL, 33.8 mmol, 2.9 equiv.) slowly. The reaction mixture was warmed up to room temperature and stirred for 17 h. The reaction mixture was filtered, and the solvent removed. The residue was extracted using diethyl ether to give off a white wax-like solid after removing the solvent under reduced pressure (10.13 g, 4.99 mmol, 91%).

^1^H NMR (500 MHz, MeOD) δ = 4.37 (m, 2H, CH_2_-OMs), 3.76 (m, 2H, CH_2_-CH_2_-OMs), 3.63 (m, 58H, PEG backbone), 3.54 (m, 2H, CH_2_-OCH_3_), 3.36 (s, 3H, OCH_3_), 3.12 (s, 3H, OSO_2_-CH_3_). ^13^C NMR (126 MHz, MeOD) δ = 72.99 (CH_2_-OCH_3_), 71.59 (PEG backbone), 71.38 (PEG backbone), 71.01 (CH_2_-CH_2_-OSO_2_-CH_3_), 70.14 (CH_2_-OSO_2_-CH_3_), 59.10 (OCH_3_), 37.61 (OSO_2_-CH_3_).

*mPEG-NH_2_**4***

Based on a procedure [20], mPEG-OMs **3** (4.54 g, 5.48 mmol) was dissolved in an aqueous 25% NH_3_ solution (40 mL) and stirred for 2 days at room temperature (r.t.) with a sealed cap. Afterwards, it was opened to air and the ammonia allowed to evaporate over the weekend. The pH of the reaction mixture was increased to 13 using 1 M NaOH and extracted with CH_2_Cl_2_ (3 × 100 mL). The combined organic phases were dried over sodium sulphate and concentrated using reduced pressure to afford a white wax-like solid (3.74 g, 8.49 mmol, 91%).

^1^H NMR (500 MHz, MeOD) δ = 3.64 (m, 58H, PEG backbone), 3.53 (m, 6H, PEG backbone), 3.36 (s, 3H, OCH_3_), 2.80 (t, J = 5.4, 2H, CH_2_-NH_2_). ^13^C NMR (126 MHz, MeOD) δ = 73.26 (CH_2_-CH_2_-NH_2_), 72.97 (CH_2_-OCH_3_), 71.56 (PEG backbone), 71.55 (PEG backbone), 71.36 (PEG backbone), 71.25 (PEG backbone), 59.10 (OCH_3_), 42.07 (CH_2_-NH_2_).

*11,11′-disulfanediyldiundecanoic acid **6a***

Disulfide **6a** was synthesized following a procedure [21]. 11-mercaptoundecanoic acid **5a** (7.21 g, 33.0 mmol, 1.0 equiv.) was dissolved in MeOH (165 mL, 5 mL per mmol acid). NaOH (0.66 g, 16.5 mmol, 0.5 equiv.), I_2_ (4.19 g, 16.5 mmol, 0.5 equiv.) and KI (0.16 g, 0.99 mmol, 0.03 equiv.) were added under stirring. The milky yellow solution was stirred for 17 h at r.t. and then decolored with saturated aq. Na_2_SO_3_ (20 mL). The solvents were removed under reduced pressure at 50 °C and the resulting aqueous slurry was dispersed in CH_2_Cl_2_ and acidified to pH 1 with 1M HCl. The aqueous phase was decanted, and the organic phase was washed with water (100 mL). The organic phase was dried over sodium sulphate and filtered. Removal of the solvent yielded a white powder (7.18 g, 100% yield).

^1^H NMR (500 MHz, CDCl_3_) δ = 2.67 (t, J = 7.3, 4H, CH_2_-SS-CH_2_), 2.29 (t, J = 7.5, 4H, CH_2_-COOH), 1.64 (m, 8H, S-CH_2_-CH_2_-(CH_2_)_6_-CH_2_-CH_2_-COOH), 1.28 (m, 24H, S-CH_2_-CH_2_-(CH_2_)_6_-CH_2_-CH_2_-COOH). ^13^C NMR (126 MHz, CDCl_3_) δ = 180.38 (COOH), 39.37 (CH_2_-SS-CH_2_), 34.19 (CH_2_-COOH), 29.51, 29.44, 29.34, 29.30, 29.13, 28.61, 24.78 (CH_2_CH_2_-COOH).

#### 2.2.2. Synthesis of the Dishells

*mPEG-NHCO-C_10_-SS-C_10_-CO_2_H **7a***

mPEG-NH_2_ **4** (2.25 g, 3.00 mmol, 1.0 equiv.) and disulfanediyldiundecanoic acid **6a** (3.91 g, 9.00 mmol, 3.0 equiv.) were stirred in bulk at 100 °C for 3 h under high vacuum. After cooling down the reaction mixture to 60 °C, the bulk mixture was diluted using methanol (50 mL) and further cooled down using cold water. The formed, white precipitation was filtered off to give a brown wax (3.00 g, 2.57 mmol, 86%).

^1^H NMR (500 MHz, MeOD) δ = 3.64 (m, 58H, PEG backbone), 3.53 (m, 6H, PEG backbone), 3.36 (s, 3H, OCH_3_), 2.69 (t, J = 7.2, 4H, CH_2_-SS-CH_2_), 2.32 (t, J = 7.4, 2H, CH_2_-COOH), 2.19 (t, J = 7.5, 2H, CH_2_-NH_2_), 1.64 (m, 8H, S-CH_2_-CH_2_-(CH_2_)_6_-CH_2_-CH_2_-COOH), 1.32 (m, 24H, S-CH_2_-CH_2_-(CH_2_)_6_-CH_2_-CH_2_-COOH). ^13^C NMR (126 MHz, MeOD) δ = 176.36 (COOH), 175.99 (NHCOOH), 72.99, 71.61, 71.59, 71.57, 71.38, 71.29, 70.62, 59.11, 51.97, 40.37, 39.79, 37.06, 34.81, 30.58, 30.57, 30.54, 30.50, 30.44, 30.36, 30.31, 30.29, 30.28, 30.20, 30.18, 29.45, 29.42, 27.03, 26.03.

*mPEG-NHCO-C_20_- CO_2_H **7b***

Docosanedioic acid **6b** (1.56 g, 4.20 mmol, 4.0 equiv.) and mPEG-NH_2_ **4** (0.79 g, 1.05 mmol, 1.0 equiv.) were stirred in bulk at 120 °C till a melt was formed and subsequently stirred under vacuum for 3 h. The bulk mixture was cooled down and MeOH was added under stirring at rt. The dispersion was repeatedly separated under centrifugation at 4000 r/min at 4 °C, and the collected supernatants were concentrated under reduced pressure. A short column chromatography of the reaction mixture (R_f_ = 0.67, MeOH/CH_2_Cl_2_ 1:9) afforded an orange solid (0.52 g, 0.46 mmol, 44%).

^1^H NMR (500 MHz, MeOD) δ = 3.64 (m, 58H, PEG backbone), 3.54 (m, 4H, PEG backbone), 3.36 (s, 3H. OCH_3_), 2.27 (t, J = 7.4, 2H, CH_2_-COOH), 2.19 (t, J = 7.5, 2H, CH_2_-NH_2_), 1.60 (m, 4H, NH-CH_2_-CH_2_-(CH_2_)_14_-CH_2_-CH_2_-COOH), 1.30 (m, 28H, NH-CH_2_-CH_2_-(CH_2_)_14_-CH_2_-CH_2_-COOH). ^13^C NMR (126 MHz, MeOD) δ = 177.74 (COOH), 177.70 (NHCOOH), 176.39, 72.97, 71.57, 71.36, 71.27, 70.61, 59.11 (OCH_3_), 40.36, 37.06, 34.96, 30.81, 30.76, 30.74, 30.73, 30.71, 30.65, 30.61, 30.47, 30.43, 30.30, 30.24, 27.04, 26.10.

#### 2.2.3. Synthesis of the Nanocarriers

General procedure: The corresponding dishell (1.5 equiv.) was dissolved in MES pH 5 buffer (50 mL per mmol hPG-NH_2_), and NHS (1.6 equiv.) was added. Cooled with an ice bath, EDCI (1.6 equiv.) was added and the reaction mixture warmed up to room temperature. hPG in water (1.0 equiv.) was added to the reaction mixture. In the case of the FITC-labelled products, hPG-NH_2_ was stirred in DMSO and FITC (3 molecules per hPG) in the dark at room temperature 2 d prior to the synthesis. After stirring for 24 h, the reaction mixture was concentrated to about 10 mL under reduced pressure and purified through either dialysis in water (benzoylated cellulose, MWCO = 14,000 g/mol, dialysis tubing by Carl Roth) or a sephadex (Cytiva) column to afford the product.

*rsCMS **1a***

General procedure. Dishell (1.40 g, 1.20 mmol), MES buffer (40 mL), NHS (0.15 g, 1.28 mmol), EDCI (0.25 g, 1.28 mmol) and hPG-NH_2_ in water (84 mg in 4.2 mL, 0.80 mmol NH_2_) afforded a yellow stock solution in water (70% yield).

^1^H NMR (500 MHz, MeOD) δ = 3.64 (m, hPG and PEG backbone), 3.36 (m, OCH_3_), 2.69 (t, J = 7.2, CH_2_-SS-CH_2_), 2.32 (t, J = 7.4, CH_2_-COOH), 2.19 (t, J = 7.5, CH_2_-NH_2_), 1.40 (m, 1H, alkyl backbone). ^13^C NMR (126 MHz, MeOD) δ = 176.32, 175.97, 72.95, 71.53, 71.33, 71.26, 70.60, 59.11, 51.99, 40.34, 39.79, 37.05, 34.80, 30.62, 30.57, 30.55, 30.52, 30.48, 30.43, 30.34, 30.29, 30.28, 30.26, 30.19, 30.19, 30.16, 29.44, 29.41, 27.02, 27.01, 26.02.

*ccCMS **1b***

General procedure. Dishell (1.40 g, 1.20 mmol), MES buffer (40 mL), NHS (0.15 g, 1.28 mmol), EDCI (0.25 g, 1.28 mmol) and hPG-NH_2_ in water (84 mg in 4.2 mL, 0.80 mmol NH_2_) afforded a yellow stock solution in water (65% yield).

^1^H NMR (500 MHz, MeOD) δ = 3.64 (m, hPG and PEG backbone), 3.36 (s, OCH_3_), 2.22 (m, alkyl backbone), 1.40 (m, alkyl backbone). ^13^C NMR (126 MHz, MeOD) δ = 178.66, 176.25, 72.93, 71.52, 71.46, 71.32, 71.23, 70.57, 59.10, 40.31, 37.02, 35.89, 30.79, 30.65, 30.48, 30.40, 30.30, 27.01, 26.46.

*FITC-labelled nanocarriers*

rsCMS-(FITC) and ccCMS-(FITC) were synthesized based on the general procedure while using hPG-NH_2_ that was stirred for 2 d with FITC (3 molecules per hPG) in dry DMSO prior to use.

#### 2.2.4. Drug Encapsulation

*Encapsulation of host molecules* via *horn sonication.* To 1 mL of a CMS stock solution in Milli-Q water, 2.5 mg of the drug was added (5 mg/mL) in order to achieve a concentration of 50 wt%. The mixture was horn sonicated for 2–5 min using an ice bath for cooling. For PhA, drug encapsulation was additionally performed by stirring the carrier with 50 wt% PhA and 100 µL acetone for 2 h, removing acetone afterwards and subsequently stirring for another 22 h. In all cases, non-encapsulated drug was removed by centrifugation at 4000 rpm for 3 min. The supernatant was stored at 4 °C in the dark.

#### 2.2.5. Characterization Methods

*Nuclear magnetic resonance (NMR).*^1^H-NMR and ^13^C-NMR spectra were acquired at room temperature by a JEOL ECP 500 and Bruker AVANCE 500. Tetramethyl silane (TMS) was used to calibrate the chemical shift δ which is reported in parts per million (ppm). The residual solvent peak was used as a reference (CDCl_3_, δ ^1^H = 7.26 ppm, ^13^C = 77.16 ppm; MeOD, δ ^1^H = 3.31 ppm, ^13^C = 49.00 ppm). The peak multiplicity is quoted as *s* = singlet, *d* = doublet, *t* = triplet, *q* = quartet and *m* = multiplet, while the coupling constants *J* are reported in Hertz (Hz). MestReNova version 14.1.1 was used for data processing. NMR spectra of the synthesized compounds are available in the supporting information.

*Gel permeation chromatography (GPC).* GPC was used to measure the size of the synthesized polymers. For GPC measurements, a Shimadzu liquid chromatograph (Kyoto, Japan) with a pump, degasser, column oven and a differential refractometer was used. The mobile phase, consisting of dimethylformamide (DMF) with 0.3% LiBr and 0.6% acetic acid, was used at a flow rate of 1 mL/min. The three PolarSil columns (PSS Polymer Standards Service GmbH, Mainz, Germany; PolarSil 8 mm × 300 mm, 100, 1000, 3000 Å with 5 µm particle size) that were used were operated at 40 °C with the refractive index (RI) detector set to the same temperature. Samples were measured at a concentration of 10 mg/mL injecting 100 µL. A polystyrene standard (PSS, Mainz, Germany) was used for calibration. LC solution software from Shimadzu was used for data acquirement and interpretation.

*Dynamic light scattering (DLS).* A Malvern Zetasizer Nano instrument (Malvern Instruments Ltd., Worcestershire, UK) equipped with a He-Ne laser (633 nm) using the backscattering mode (detector angle 173°) was used for DLS measurements. To measure the hydrodynamic diameter (D_H_), the CMS nanocarriers were dissolved in Milli-Q water or PBS mixed by a vortex shaker for 2 min, and 100 µL of the solution were added to a disposable Plastibrand^®^ micro cuvette (Brand GmbH + Co KG, Wertheim, Germany) with a round aperture. The measurements were performed at 25 °C, equilibrating the system at this temperature for 120 s. The Zetasizer DLS software (Malvern Instruments Ltd., Worcestershire, UK) was used to determine the size distribution by intensity and volume.

*Cyclic voltammetry.* An autolab PGSTAT302N potentiostat using a three-electrode configuration was used for cyclic voltammetric measurements. Samples were dissolved in dry DMSO, purging nitrogen through it before measuring. Cyclic voltammetric and differential pulse voltammetry (DPV) measurements were performed at a scanning speed of 100 mV/s using glassy carbon as a working electrode, a platinum wire as a counter electrode and a silver wire as a pseudo reference electrode. Raw data were analyzed using Nova 1.5 by Metronohm and figures were plotted using Igor Pro.

*High-performance liquid chromatography (HPLC).* A Knauer Smartline-HPLC system with an internal UV absorption detector (λ = 254 nm), equipped with a Gemini RP C18 column (Phenomenex, 250 nm × 4.6 mm, particle size: 5 µm) was used for isocratic HPLC measurements. Acetonitrile was used as a mobile phase at a 1.0 mL/min flow rate. Data analysis was performed using Chromgate software (Knauer, Berlin, Germany). For the determination of the drug loading content (DLC) of rapamycin and dexamethasone via HPLC, 50 µL of the drug@CMS solutions were freeze-dried and then dissolved in 300 µL acetonitrile. The concentration of the drug in solution was determined using a calibration curve of rapamycin in acetonitrile (concentration range: 0.5–0.015625 mg/mL). Calibration curves were freshly prepared and measured prior to sample measurements (Figures S15 and S16).

*Ultraviolet and visible spectroscopy (UV–Vis).* An Agilent Cary 8454 UV-Visible spectrophotometer using Suprasil^®^ from Hellma analytics (Hellma GmbH & Co. KG, Müllheim, Germany) or Spectrosil^®^ (Carl Roth, Karlsruhe, Germany) half-micro quartz cuvettes were used for UV–Vis measurements. Data were collected using the UV-Vis ChemStation Software version B.05.02 (Agilent Technologies, Santa Clara, CA, USA). For the determination of the DLC through UV–Vis, 50 µL of the drug-encapsulated solution were freeze-dried and dissolved in either acetonitrile for Nile red (NR), or in acetone for mTHPP and PhA. The concentration in the sample was determined using the Lambert–Beer law.

*Fluorescence spectroscopy.* Measurements were performed on a Scinco S-3100 spectrometer at 37 °C, maintained with a Haake F3 thermostat. Measurements were conducted using Suprasil^®^ (Hellma Analytics) half-micro quartz cuvettes with a 4 × 10 mm light path and stopper, and an excitation and emission band width of 5 nm was chosen for the measurements. For the release of NR@rsCMS with TCEP, 100 µL of a 5 mg/mL NR@rsCMS solution in Milli-Q water were dried and dissolved in 10-fold concentrated PBS. Then 10 mM TCEP was added and the pH was set to 6.4 with 310 µL 1M KOH. The fluorescence measurement at 37 °C was started directly after dissolution, and spectra were taken every 5 min for 24 h. The same conditions were used for the release study of NR@ccCMS while measuring the fluorescence spectrum before and after 24 h. For the release of NR@rsCMS with 10 mM GSH, the solution was prepared correspondingly, adding 10 mM GSH and keeping a pH of 7.4. The fluorescence measurement at 37 °C was started directly after dissolution, and spectra were taken every 30 min for 24 h. For the GSH release study with a mixed solution, the sample was shaken at 37 °C, facilitating a mixing of the solution, and taken out at regular intervals to measure the fluorescence spectrum for 24 h. Control release experiments were conducted with rsCMS in 10-fold concentrated PBS, but in the absence of any reducing agent. The respective fluorescence measurements at 37 °C were started directly after dissolution, and spectra were taken every 30 min for 24 h.

#### 2.2.6. Skin Penetration Experiments

Skin samples from healthy donors undergoing plastic surgery were obtained after informed consent. The study was conducted after approval by the Ethics Committee of the Charité—Universitätsmedizin Berlin (approval EA1/135/06, renewed in January 2019) and in accordance with the Declaration of Helsinki guidelines. A few hours after the surgery, the skin was cleaned and examined microscopically, intact skin areas were cut out, subcutaneous fat was removed, and skin pieces of 1.5 × 1.5 cm were prepared. Skin pieces were placed on trans-well inserts with an 8 µm pore membrane (Cell Culture Inserts, BD Falcon™, Durham, NC, USA) and these were placed in a 6-well plate filled with 2 mL RPMI-1640 medium (PAA, Heidelberg, Germany), supplemented with 10% fetal calf serum (FCS, PAA, Heidelberg, Germany), 100 I.E./mL of penicillin and 100 g/mL of streptomycin. The tested formulations, 40 µL of 5 mg/mL CMS nanocarrier suspensions in 2.5% HEC gel, were applied on approximately 1 cm^2^ of skin, leaving untreated margins. Samples were then incubated for 24 h in an incubator at 37 °C, 5% CO_2_, 100% humidity. Skin samples treated with 2.5% HEC gel only served as controls. After incubation, the non-penetrated material on the top of skin was removed with a cotton swab; the central treated part of the skin samples was cut with an 8 mm punch biopsy and frozen in liquid nitrogen. Skin sections of 5 µm in thickness were prepared with a cryotome (Frigocut 2800 N, Leica, Bensheim, Germany). Skin from three donors was treated. From each skin sample, at least 20 sections were prepared and at least 20 pictures were taken with a confocal fluorescence scanning microscope (LSM 700 (Zeiss, Jena, Germany)). At least 30 images per CMS sample and control were analyzed using ImageJ software version 1.47 (National Institute of Health, Bethesda, MD, USA). The mean fluorescence intensity (MFI) of SC, viable epidermis (VE) and dermis (D) was calculated for each section. Sample values were subtracted from the MFI of the respective controls and normalized with respect to the mTHPP loading percentage of the respective CMS nanocarrier. Averages and standard errors were calculated, and graphics were prepared using Office Excel (Microsoft Corp., Redmond, WA, USA). Statistical analysis was done using one-way analysis of variance (ANOVA) followed by comparison of two groups with a Student’s *t*-test.

## 3. Results and Discussion

### 3.1. Synthesis of the CMS Nanocarriers and Their Building Blocks

**Scheme 1:**
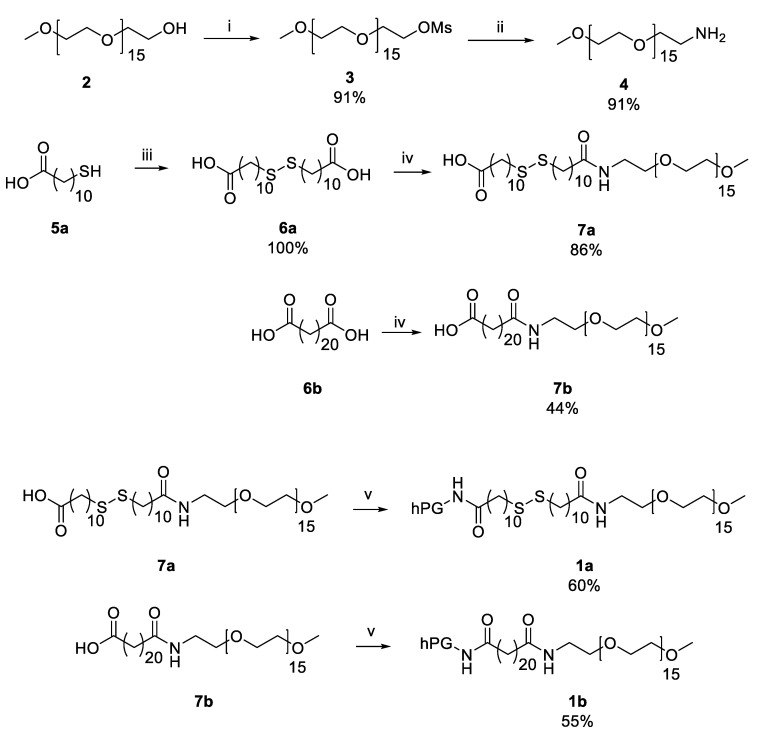
Synthesis of redox-sensitive CMS (rsCMS) **1a** and non-redox sensitive nanocarrier (ccCMS) **1b**. The following conditions were used: (i) NEt_3_, MsCl, CH_2_Cl_2_, 0 °C– room temperature (r.t.), 17 h, (ii) NH_3_ (25%) aq, 2 d, (iii) NaOH, I_2_, KI, MeOH, 17 h, r.t., (iv) PEG_750_-NH_2_, bulk, high vacuum, 120 °C, 3 h, (v) (1) *N*-hydroxysuccinimide, ethyl(dimethylaminopropyl)carbodiimide, 2-Morpholinoethanesulfonic acid pH 6, 0 °C, r.t. (2) hPG-NH_2_, 24 h, r.t.

The CMS nanocarriers were synthesized by a convergent synthetic approach, combining two pre-synthesized building blocks (Scheme 1).

The outer shell, mPEG-NH_2_ **4**, was obtained by mesylating the hydroxy group of mPEG-OH **2** to mPEG-OMs **3** and subsequent amination. Both reactions were performed with a yield of 91%. Disulfide **6a** was obtained through oxidation of mercaptoundecanoic acid **5a** with a yield of 100%. Amidation of mPEG-NH_2_ **4** with a four-fold excess of disulfide **6a** through condensation under vacuum led to dishell **7a** with a yield of 86%. Its non-sulfuric counterpart was synthesized in a similar fashion affording 44% of dishell **7b**. The double shell building blocks were conjugated to the core building block hPG-NH_2_ through amide bond formation in aqueous medium. Thereby, dishell **7a** led to the rsCMS nanocarrier **1a** with a yield of 70% and dishell **7b** led to the non-reduction-sensitive ccCMS nanocarrier **1b** with a yield of 65%. Purification via dialysis in water led to a CMS with narrow polydispersity indexes (PDIs) between 1.1 and 1.2 (Table 1). NMR spectra of all materials are depicted in the supplementary information (Figures S1–S14). The FITC-labelled nanocarriers were synthesized accordingly while stirring hPG-NH_2_ with FITC (three molecules per hPG) in DMSO in the dark at room temperature 2 d prior to the synthesis.

The molar mass of the synthesized CMS nanocarriers depends on the amount of conjugated double shell, reflected by the degree of functionalization (DF). Assuming a full conversion of all 95 amine groups of hPG-NH_2_ and a resulting DF of 100%, all nanocarriers have a theoretical molar mass of above 100 g/mol (Table 1). GPC and ^1^H NMR (NMR-based calculation of *M*_n_ in SI) indicated a lower degree of functionalization with lower molar masses. Reactions between the double shell **7b** and the highly aminated hPG result in poor conversions, reflected by the mediocre DF value of 67% for ccCMS **1b**. The rsCMS **1a**, on the other hand, resulted in a higher conversion rate with a DF of 89%. Despite the deviant molar mass of rsCMS **1a** and ccCMS **1b**, GPC analysis gave comparably similar *M*_n_. This has been observed before and shows the frequent limitation of GPC performed with linear standards in the analysis of hyperbranched polymers [22,23]. The PDI value of both carriers varies from 9.3 nm for rsCMS **1a** to 14.3 nm for ccCMS **1b**. The latter is based on aggregation and has also been reported for comparable structures [24]. While the PDI value of the GPC measurement indicates a narrow monodisperse distribution, the PDI value indicates a moderate polydispersity, which presumably stems from formed aggregations.

### 3.2. Redox Potential of rsCMS **1a**

To investigate the nature of the redox-sensitive environment of the rsCMS carrier **1a**, cyclic voltammetric measurements were performed to compare the reductive potential towards commonly used reducing agents. As reducing agents, TCEP and GSH, which are commonly evident in skin tissues, were used. Additionally, DPV measurements were performed to determine the redox potential.

**Figure 1:**
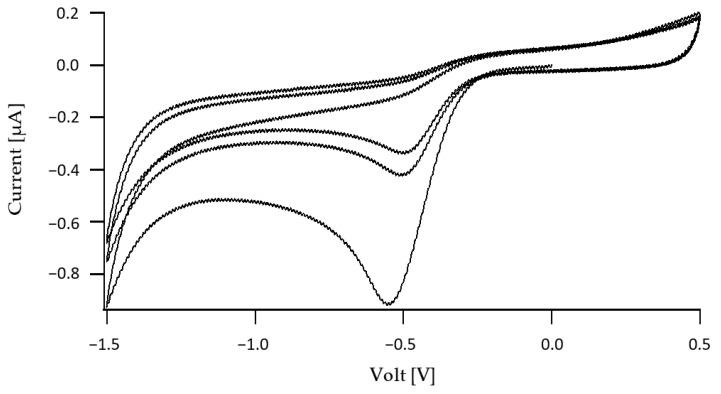
Cyclic voltammetric measurement of rsCMS **1a** in dry DMSO at a scan rate of 100 mV/s, three rounds.

The rsCMS carrier **1a** showed a reducing potential at −0.51 V and an oxidation potential at −0.26 V (Figure 1), which stem from the reduction and subsequent oxidation of its disulfide bonds. DPV measurements gave an overall redox potential of −0.44 V. The structurally similar disulfide **6a** showed a reduction potential at −0.55 V and an oxidation potential at −0.38 V and an overall redox potential of −0.51 V, indicating a less pronounced reducibility (S17).

TCEP shows a reduction potential of −1.65 V while the oxidation potential is hidden under the voltammetric curve (Figure S18). Its overall redox potential was determined as −1.62 V. The redox process stems from the oxidation and subsequent reduction of the phosphor moiety. As to be expected, GSH shows a higher reduction potential at −0.75 V and an oxidation potential at −0.65 V, being a milder reducing agent (Figure S19). For the actual measurement, its oxidized counterpart GSSG was used, as the detection limit of GSH is rather high and its use is limited due to its low solubility in DMSO. Its redox potential was determined as −0.70 V. Its redox process can be classified as the thiol oxidization and disulfide cleavage. Having a higher reduction potential than rsCMS **1a**, both compounds function as reducing agents for rsCMS **1a**.

Being measured under the same conditions, the redox potentials of TCEP, GSH and rsCMS **1a** can be compared. Changes in the condition, such as different solvents, choice of electrodes or pH dependence can lead to somewhat large changes in the redox potential, making direct comparisons with the literature unreliable [25]. An overview of the redox potentials is depicted in Table 2.

### 3.3. In Vitro Stimulus-Triggered Release of NR from CMS Nanocarriers

Stimulus-triggered release of drugs from CMS nanocarriers was performed for a proof of concept using NR as a model drug. The fluorescent dye NR has a poor water solubility. Encapsulated into a nanocarrier, its water solubility can be enhanced, which results in a higher NR fluorescence intensity compared to an aqueous solution of the dye. Once released from the nanocarrier, NR either forms aggregates, or precipitates due to its low solubility in water. This results in a reduced fluorescence intensity of the aqueous medium. The dye release can therefore be measured through the fluorescence intensity of the suspension using the wavelength maximum of NR emission [26].

**Figure 2:**
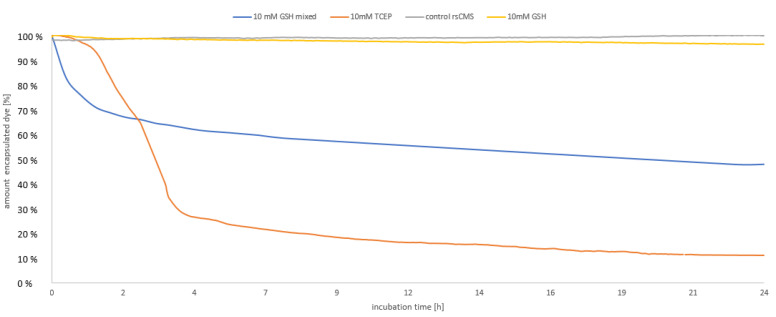
Kinetics of stimulus-triggered release of Nile red (NR) from rsCMS **1a** measured with fluorescence spectroscopy over 24 h; NR release was followed by fluorescence intensity decay at NR emission maximum wavelength.

The redox environment of a cell and its redox state in a biological context are still not fully understood to this day [27,28]. The GSH/GSSG couple is the most abundant redox couple in a cell. Their ratio of 100:1 is constantly maintained by enzymes such as *γ*-glutamylcysteine synthetase or glutathione synthetase [27]. The cytosolic concentration of GSH varies between 1 and 11 mM. For a proof-of-concept study, a concentration of 10 mM GSH was chosen (Figure 2). To balance the acidifying properties of GSH, a 10 mM PBS solution (pH 7.4) was used. The fluorescence measurement of the GSH-triggered release under incubation without shaking shows a fluorescence decrease of 4% within 24 h (Figure 2, yellow line). Having a higher molecular weight, the accessibility of GSH to the disulfide bond is limited, resulting in a low drug release. To facilitate the accessibility of the reduction site, the same release experiment was performed under continuous shaking, measuring the release within 24 h (Figure 2, blue line). In this case, a significant drug release was evident, with 36% of NR released within 4 h, and 52% released after 24 h. The TCEP-triggered release led to a significant release under incubation without shaking, with a 70% release after 4 h and an overall release of 90% within 24 h (Figure 2, orange line). The sigmoidal shape of the release curves indicates an auto-accelerated release process as the reducing agents can access the disulfides more easily once the outer shell is cleaved [29,30,31].

As control reactions, NR release from the rsCMS nanocarrier without reducing agents (Figure 2, grey line) as well as NR release from the ccCMS nanocarrier with TCEP (Figure S20) were measured. As expected, both control reactions showed no decrease in fluorescence intensity, i.e., no triggered NR release.

The reduction of rsCMS **1a** by TCEP leads to a partial degradation of the nanocarrier and possible crosslinking. After 24 h of TCEP incubation, the previously clear sample became turbid, and DLS measurement of the filtered nanocarrier sample showed only fragments with a size less than 1 nm. Reduction by GSH, limited by the low fraction of reductive, non-oxidized GSH, as discussed above, was observed by following the methylene signal adjacent to disulfide at 2.8 ppm shifting to 2.6 ppm, assigned to the methylene group of the alkyl inner shell, adjacent to thiol (Figure S21).

In general, cleavage of disulfide-containing polymers by reducing agents, such as dithiothreitol, has been shown by methods such as DLS and GPC measurements [32,33].

### 3.4. Penetration of rsCMS-(FITC) and ccCMS-(FITC) Nanocarriers and Diffusion of the Loaded mTHPP in Ex Vivo Human Skin

**Figure 3:**
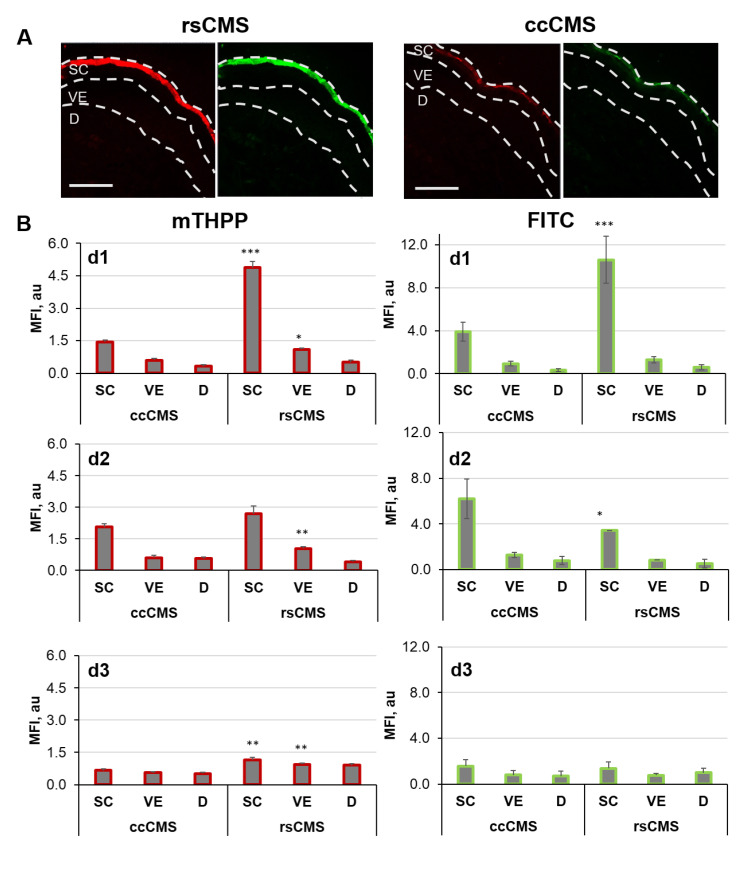
Penetration of ccCMS **1b** and rsCMS **1a** nanocarriers in ex vivo human skin and delivery of the loaded dye meso-tetra (*m*-hydroxyphenyl) porphyrin (mTHPP). (**A**) Representative images of skin sections from rsCMS **1a** and ccCMS **1b** nanocarrier-treated samples. Bars = 50 µm. (**B**) Summary of the mean fluorescence intensity (MFI) and standard errors from three different donors (d1-d3). Statistical analysis was performed to compare rsCMS **1a** values with those of ccCMS **1b** nanocarriers in the same skin layers. The stars show significant differences (* *p* < 0.05; ** *p* < 0.01; *** *p* < 0.001).

In order to test the drug delivery ability of the investigated nanocarriers, ex vivo human skin was topically treated with a 2.5% HEC gel formulation of fluorescent CMS nanocarriers (Figure 3). The penetration of the nanocarriers could be detected by the covalently bound FITC (green), whereas the nanocarrier delivery properties could be tested by measuring the fluorescence of the loaded mTHPP dye (red). In Figure 3A, representative images of skin sections from rsCMS and ccCMS samples are shown. Pictures of samples and controls were taken using the same microscope and camera settings. After normalization to the amount of loaded dye, the highest penetration rate was detected for the rsCMS samples. While the nanocarriers were observed in the SC only, a weak but significant mTHPP signal was also appreciable in the VE and D. The analysis of the pictures (at least 30 per sample) was performed using the image processing software ImageJ. Low fluorescence signals were detected for the untreated control skin in all three skin layers: SC, VE and D. The average background fluorescence of controls was subtracted from sample values before averages and normalized values were calculated. Figure 3B shows the summary of the fluorescence intensities for the three different donors.

Acceptable variations between the skin penetration results obtained from different donors were found. The highest fluorescence intensities were detected in the SC, both for mTHPP and FITC signals. Small but detectable amounts of fluorescence signal were also detected in the VE and D. Although in the VE no differences between the FITC signals of the two nanocarriers could be measured, a higher mTHPP signal was detected for rsCMS **1a** in the VE as compared to the ccCMS **1b** nanocarrier. The higher mTHPP signal in the VE of rsCMS **1a** samples can be explained by the interaction of the rsCMS **1a** nanocarriers with the SC reductive environment, the triggered dye release and the consequent positive effect on dye diffusion to the VE. In general, these results confirm the possibility to use a reduction-initiated drug release as trigger for topical drug delivery.

### 3.5. Drug-Loading Capacity

The drug-loading performance of the CMS nanocarriers was screened using the fluorescent dyes NR, mTHPP and PhA, as well as the anti-inflammatory drugs dexamethasone and rapamycin.

NR has a lower log *P* value, while porphyrins are generally very hydrophobic and are used to mimic very hydrophobic drugs [34,35]. mTHPP itself is a hydrophobic drug used as a photosensitizer in the treatment of head and neck cancer [35]. Dexamethasone and rapamycin were chosen as examples for hydrophobic drugs with low and high molecular weight, respectively. Hence, the DLC of host molecules with a broad range of water solubility was depicted.

The encapsulation of NR, dexamethasone, rapamycin, mTHPP and PhA was achieved through horn sonicating the CMS nanocarrier suspension with the drug for 2 min to achieve a DLC of 1-3 weight percent (wt%), as depicted in Table 3. There is no trend evident regarding the DLC based on the hydrophobicity or molecular weight of the drug. Increasing the horn-sonicating time for mTHPP samples led to an increased DLC from 1 wt% to 2–3 wt%. Stirring PhA with traces of acetone and removing acetone prior to the removal of excess non-encapsulated drug even increased the DLC from 2 wt% to 6 wt%, proving it to be the most effective encapsulating method. There is no visible difference in the DLC based on the carrier used. DLCs in a similar range were observed for CMS systems with similar structures [24,36].

The drug loading was comparable despite the hydrophobicity of the used drug, while different loading methods had an impact of the DLC. The final DLC could have some effect on the speed of drug release, e.g., if the carrier has a higher loading, more drug molecules might be located on the outer shell and thus be released more rapidly once the carrier reacts with reducing agents. Higher DLCs could also have an impact on the in vivo performance, as higher loading is often associated with higher drug delivery across the skin barrier. Additionally, for the same effect of the drug, less nanocarrier will be needed.

## 4. Conclusions

The synthesis of rsCMS **1a** and ccCMS **1b** resulted in chemically comparable CMS nanocarriers with sizes below 20 nm and narrow molecular weight distributions. The reduction potential of the disulfide of rsCMS **1a** was determined as 0.23 V through cyclic voltammetric measurements. Having a lower reduction potential, TCEP and GSH were proven to be adequate as reducing agents.

The proof-of-concept in vitro study supported the hypothesized triggered release of the fluorescent model drug NR, analyzed by time-dependent fluorescence spectroscopy measurements. The reduction-triggered release of NR from rsCMS **1a** was shown with GSH, resulting in a drug release of 52% within 24 h, while the release with TCEP was more pronounced and led to 90% release of the encapsulated dye within 24 h.

The ex vivo skin experiments served as a proof of concept to show the applicability of this concept for dermal drug delivery. With the SC being rich in GSH and other thiol groups, it acts as a trigger for drug delivery once the nanocarriers have penetrated the SC. The chosen macrocyclic dye has similar molecular weight and hydrophilicity (log *P*) to macrolide drugs like rapamycin.

A DLC of generally 1–6 wt% was achieved for drugs with various molecular weights and water solubility. Thus, these results show the feasibility of the proposed concept for a triggered dermal drug delivery based on the redox potential of the target tissue and great potential for macrolide drugs.

## Figures and Tables

**Table 1:** Characterization data for redox and control CMS nanocarriers.

CMS	*M*_n_, Theor. ^1^ (g/mol)	*M*_n_, NMR ^2^ (g/mol)	DF, NMR ^2^ (%)	*M*_n_, GPC ^3^ (g/mol)	PDI ^3^	D_H_, vol ^4^ (nm)	PDI ^4^
rsCMS **1a**	118.6 kDa	106 kDa	89	62.7 kDa	1.1	9.3	0.6
ccCMS **1b**	112.5 kDa	75 kDa	67	48.5 kDa	1.1	14.3	0.5

^1^ Assuming 100% conversion; ^2^ determined via ^1^H NMR; ^3^ determined via Gel permeation chromatography (GPC), polydispersity index (PDI) = *M*_n_/*M*_w_; ^4^ determined via dynamic light scattering (DLS), PDI = (std dev/mean)^2^.

**Table 2:** Redox potentials of tris (2 carboxyethyl)-phosphine (TCEP) and glutathione (GSH), disulfide **6a** and rsCMS **1a**.

Potential	TCEP	GSH	Disulfide 6a	rsCMS 1a
Reduction potential ^1^	−1.65 V	−0.75 V	−0.55 V	−0.51 V
Oxidation potential ^1^	Not detectable	−0.65 V	−0.38 V	−0.26 V
Redox potential ^2^	−1.62 V	−0.70 V	−0.51 V	−0.44 V

^1^ Measured through cyclic voltammetry; ^2^ measured through differential pulse voltammetry (DPV) measurements.

**Table 3:** Drug loading content (DLC) of rapamycin, mTHPP and Pheophorbide A (PhA) by rsCMS **1a** and ccCMS **1b**.

Drug	Log *P*	M_n_ (g/mol)	DLC in rsCMS 1a	DLC in ccCMS 1b
Dexamethasone	1.8 [37]	392.46	2 wt% ^1^	2 wt% ^1^
NR	3–5 [34]	318.37	1 wt% ^1^	1 wt% ^1^
Rapamycin	4.3 [38]	914.17	3 wt% ^1^	2 wt% ^1^
mTHPP	~10–12 [39]	678.73	1 wt% ^1^, 2 wt% ^2^	1 wt% ^1^, 3 wt% ^2^
PhA	~10–12 [39]	592.68	2 wt% ^1^, 6 wt% ^3^	2 wt% ^1^, 6 wt% ^3^

^1^ Horn sonication of carrier–drug solution for 2 min; ^2^ horn sonication of carrier–drug solution for 5 min; ^3^ stirring the carrier–drug solution for 17 h with acetone in water.

## Data Availability

Data is contained within the article or supplementary material.

## Supplementary Materials

The following are available online at https://www.mdpi.com/1999-4923/13/1/37/s1, Figure S1. ^1^H NMR of mPEG-OMs **3**, Figure S2. ^13^C NMR of mPEG-OMs **3**, Figure S3. ^1^H NMR of mPEG-NH_2_ **4**, Figure S4. ^13^C NMR of mPEG-NH_2_ **4**, Figure S5. ^1^H NMR of disulfide **6a**, Figure S6. ^13^C NMR of disulfide **6a**, Figure S7. ^1^H NMR of dishell **7a**, Figure S8. ^13^C NMR of dishell **7a**, Figure S9. ^1^H NMR of dishell **7b**, Figure S10. ^13^C NMR of dishell **7b**, Figure S11. ^1^H NMR of rsCMS **1a**, Figure S12. ^13^C NMR of rsCMS **1a**, Figure S13. ^1^H NMR of ccCMS **1b**, Figure S14. ^13^C NMR of ccCMS **1b**, Figure S15. Calibration curve of dexamethasone, Figure S16. Calibration curve of rapamycin, Figure S17. Cyclic voltammetric measurements of disulfide **6a** in dry DMSO at a scan rate of 100 mV/s, six rounds, Figure S18. Cyclic voltammetric measurements of TCEP in dry DMSO at a scan rate of 100 mV/s, two rounds, Figure S19. Cyclic voltammetric measurements of GSH in dry DMSO at a scan rate of 100 mV/s, three rounds, Figure S20. Fluorescence measurement of NR-encapsulated ccCMS incubated with TCEP before and after 24 h, Figure S21. Stacked ^1^H NMR spectra of interval measurement, 80 scans per measurement; incubation with 10 mM GSH solution in PBS pH 7.4 at 37 °C; ratio of 2.8 ppm (CH_2_-SS-CH_2_) vs. 2.6 ppm (CH_2_-SH).
