# Supplementary material for: Redox-Responsive Nanocarrier for Controlled Release of Drugs in Inflammatory Skin Diseases
Source: Pharmaceutics. 2020 Dec 29;13(1):37. doi: 10.3390/pharmaceutics13010037 (PMC7823658; doi:10.3390/pharmaceutics13010037)
Supplement: Supplementary file 1 [file pharmaceutics-13-00037-s001.pdf]

pharmaceutics

Article

Redox-Responsive Nanocarrier for Controlled Release of Drugs in Inflammatory Skin Diseases

Keerthana Rajes [1], Karolina A. Walker [1,*], Sabrina Hadam [2], Fatemeh Zabihi [2], Fiorenza Rancan [2], Annika Vogt [2] and Rainer Haag [1,*]

1. NMR spectra

Figure S1. ^{1}H NMR of mPEG-OMs **3**.

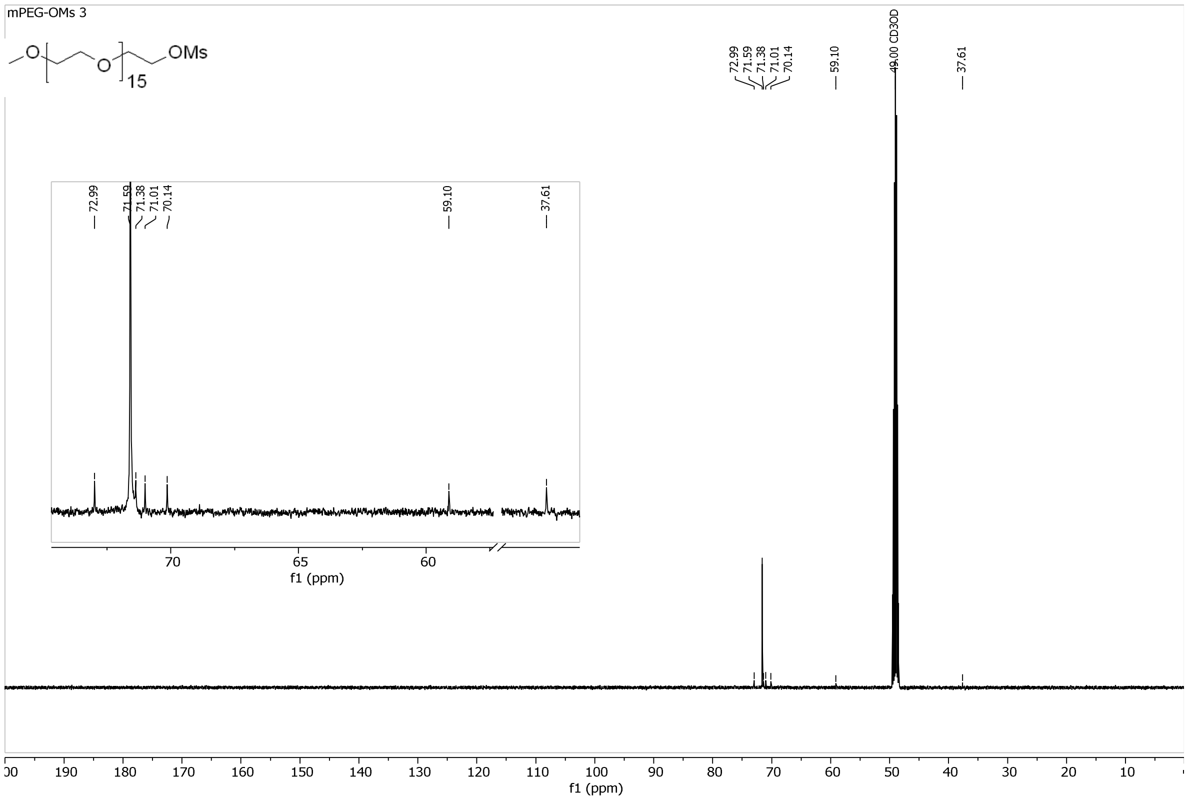

Figure S2. ^{13}C NMR of mPEG-OMs **3**.

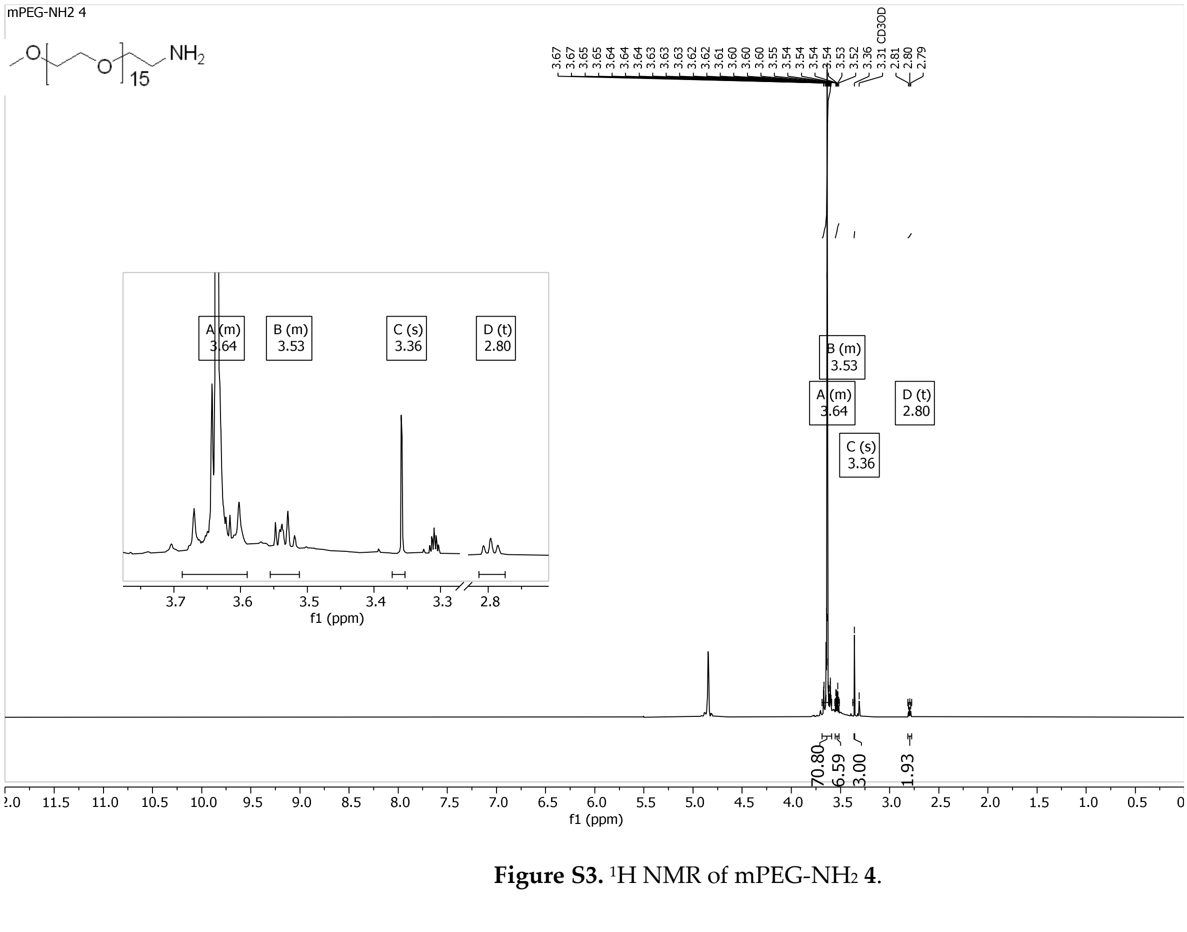

Figure S3. ^{1}H NMR of mPEG-NH_2 **4**.

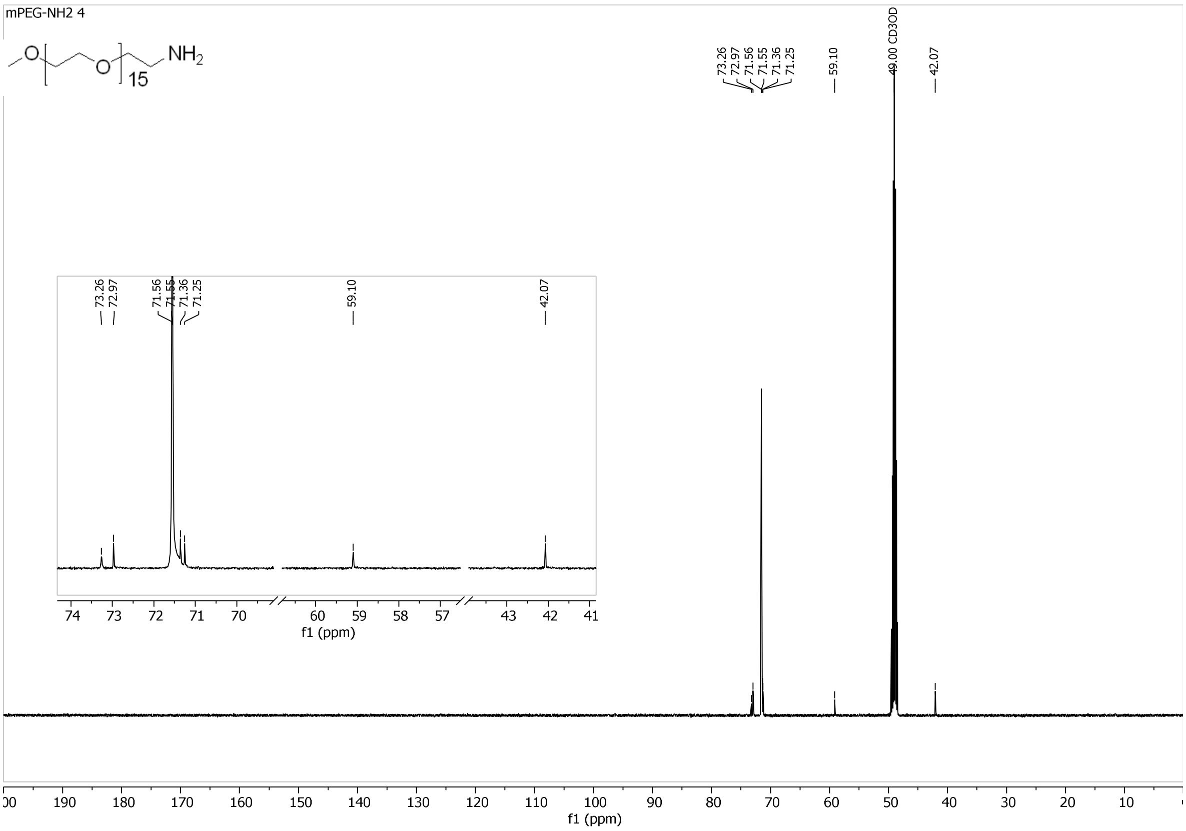

Figure S4. ^{13}C NMR of mPEG-NH$_2$ **4**.

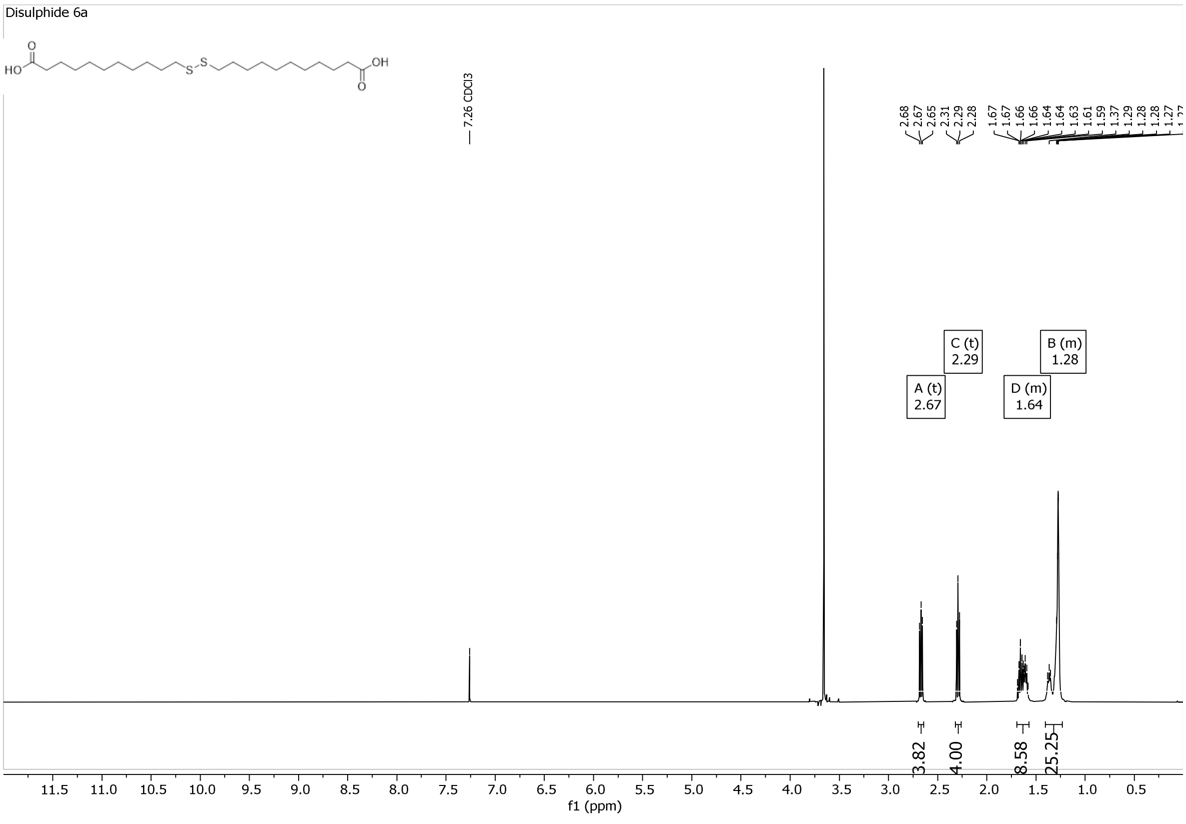

Figure S5. ^{1}H NMR of disulfide **6a**.

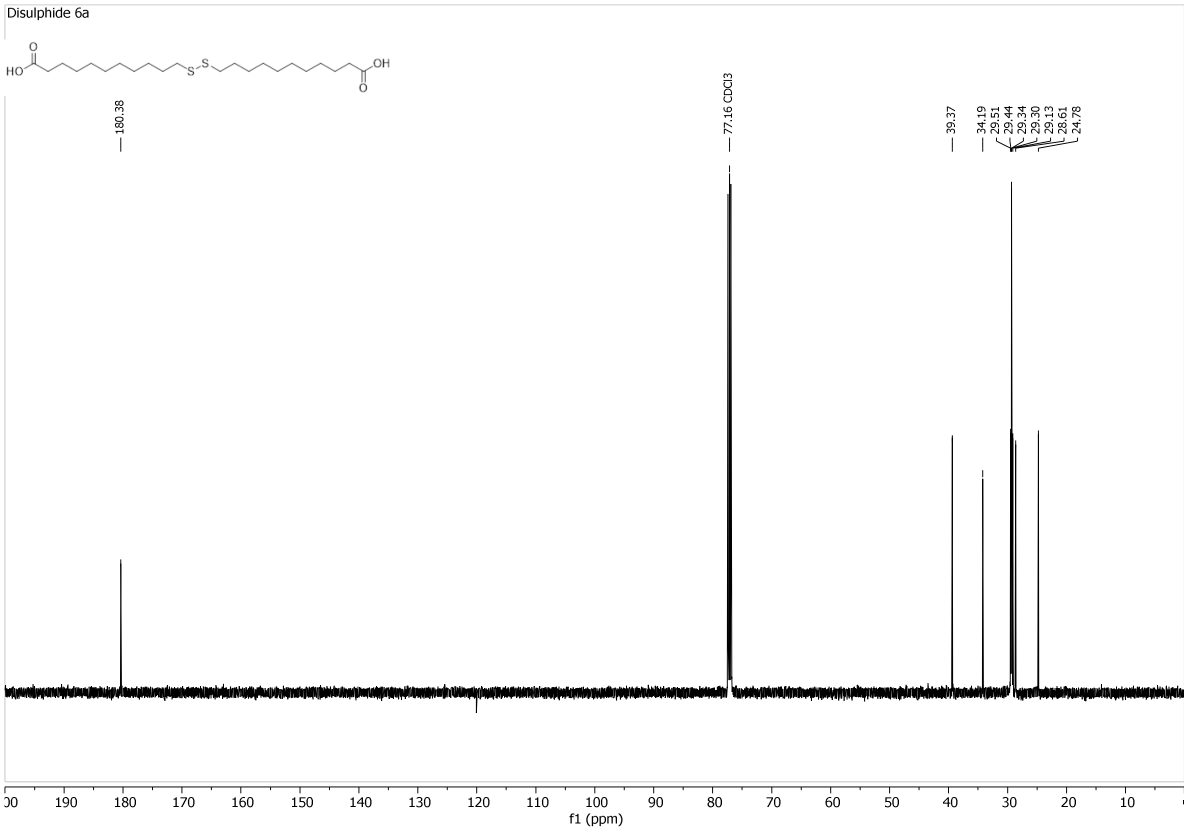

Figure S6. ^{13}C NMR of disulfide **6a**.

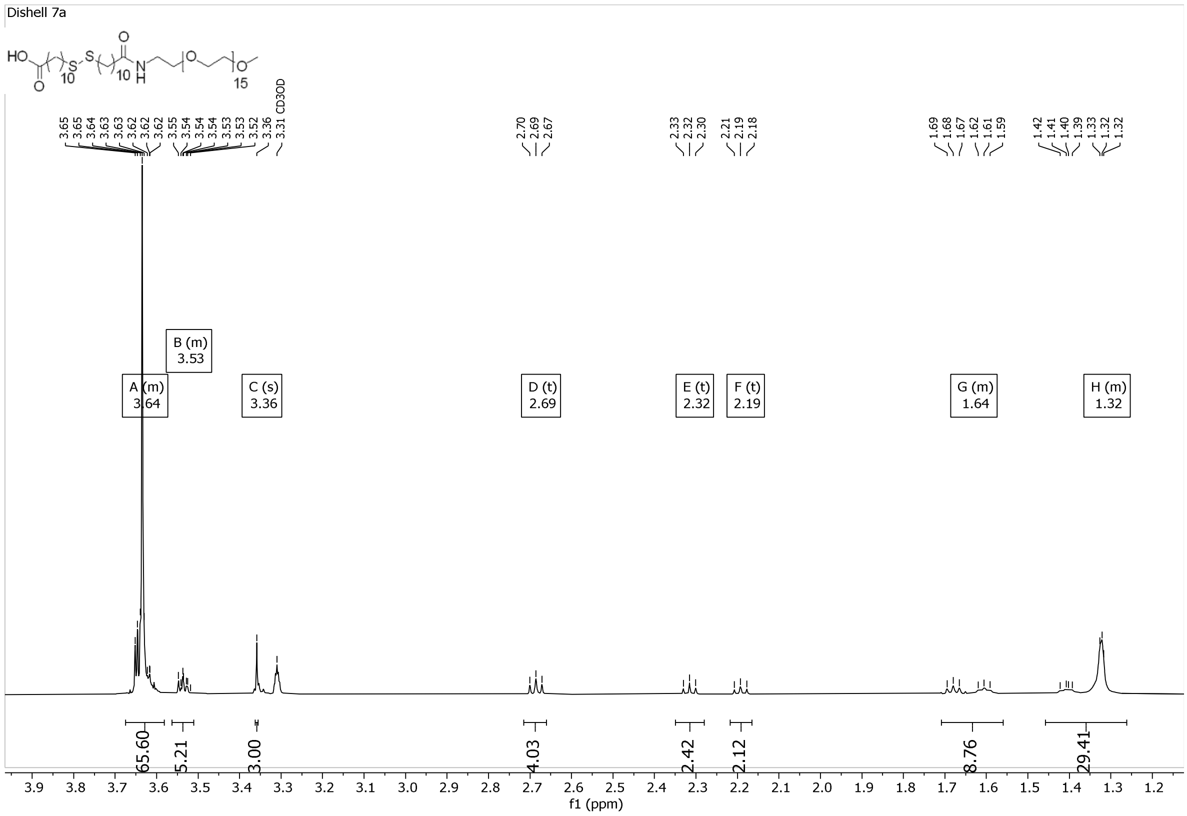

Figure S7. ^{1}H NMR of dishell **7a**.

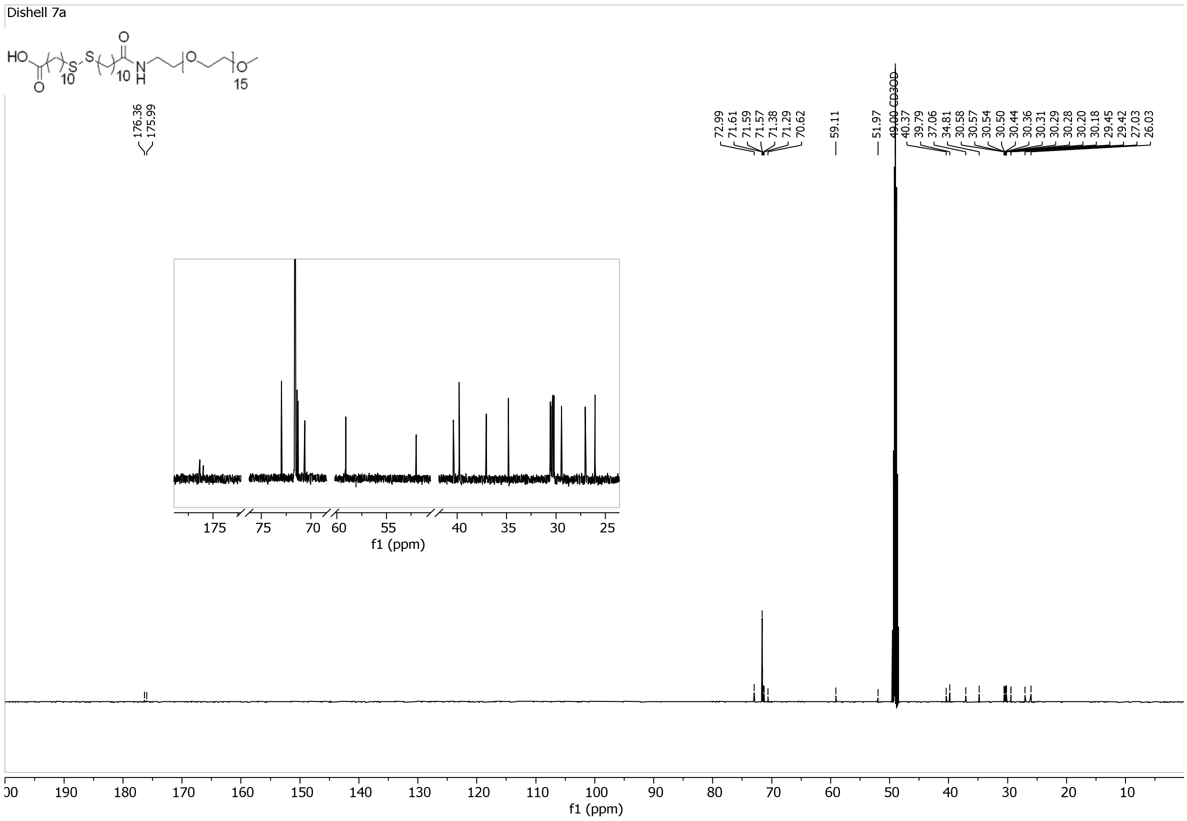

Figure S8. ^{13}C NMR of dishell **7a**.

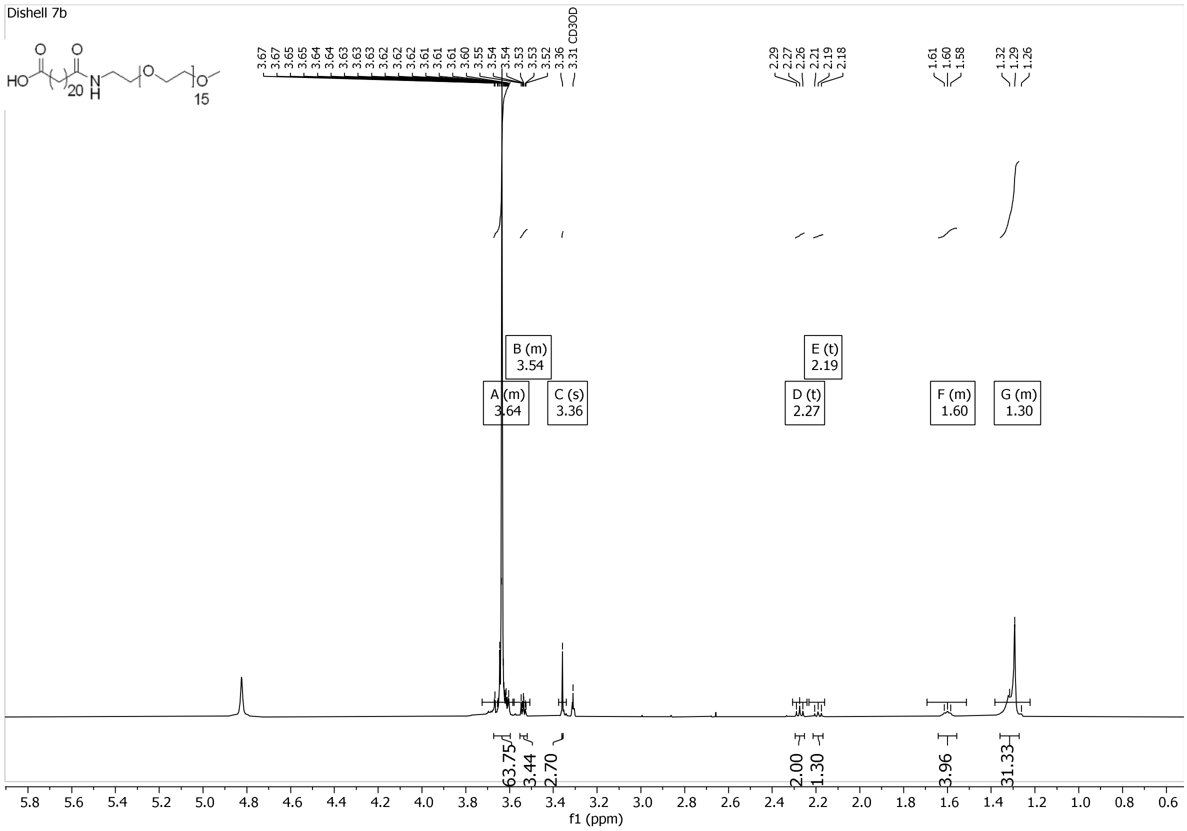

Figure S9. ^{1}H NMR of dishell **7b**.

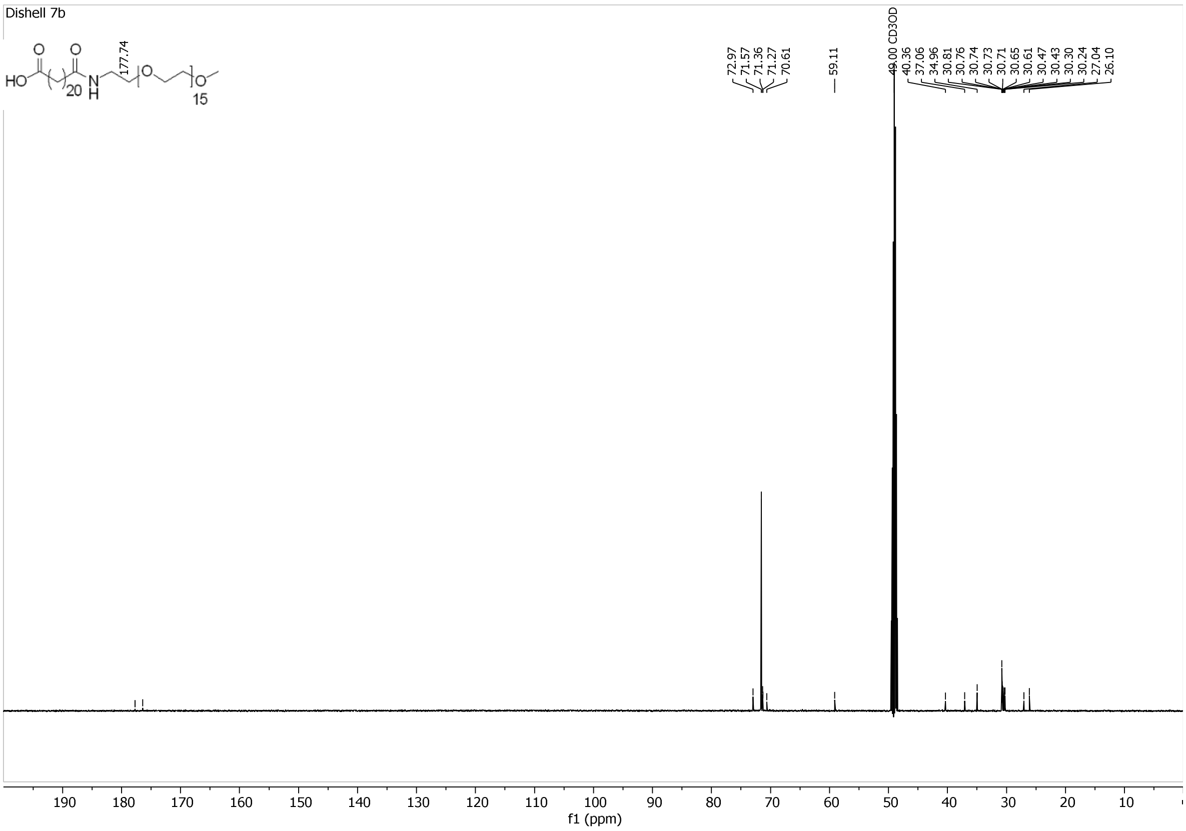

Figure S10. ^{13}C NMR of dishell **7b**.

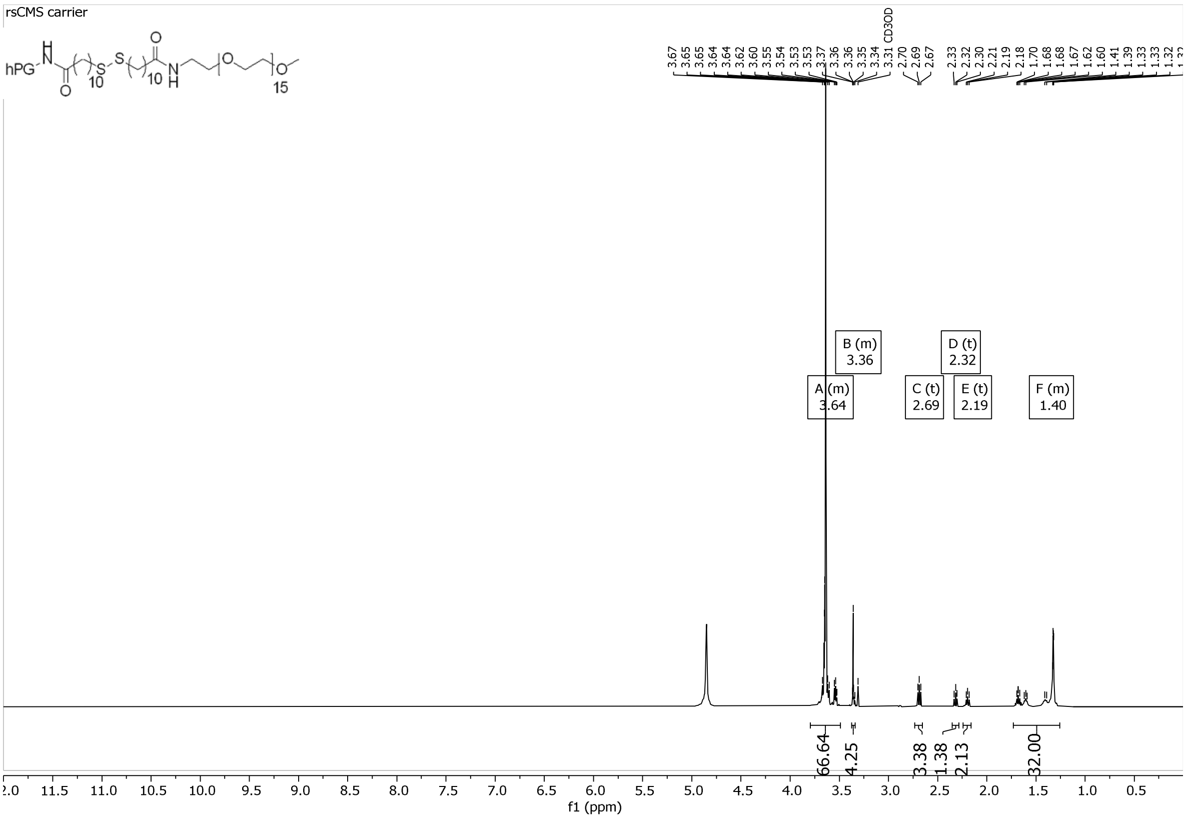

Figure S11. ^{1}H NMR of rsCMS **1a**.

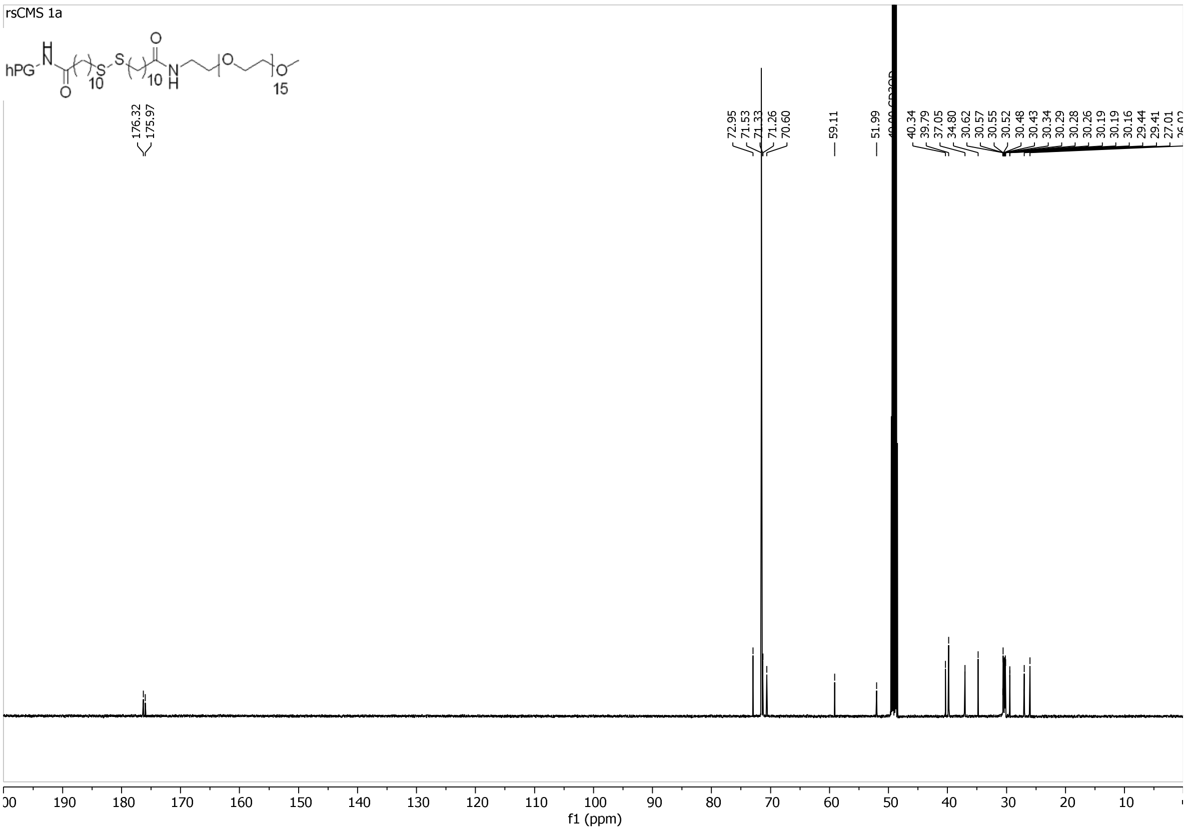

Figure S12. ^{13}C NMR of rsCMS **1a**.

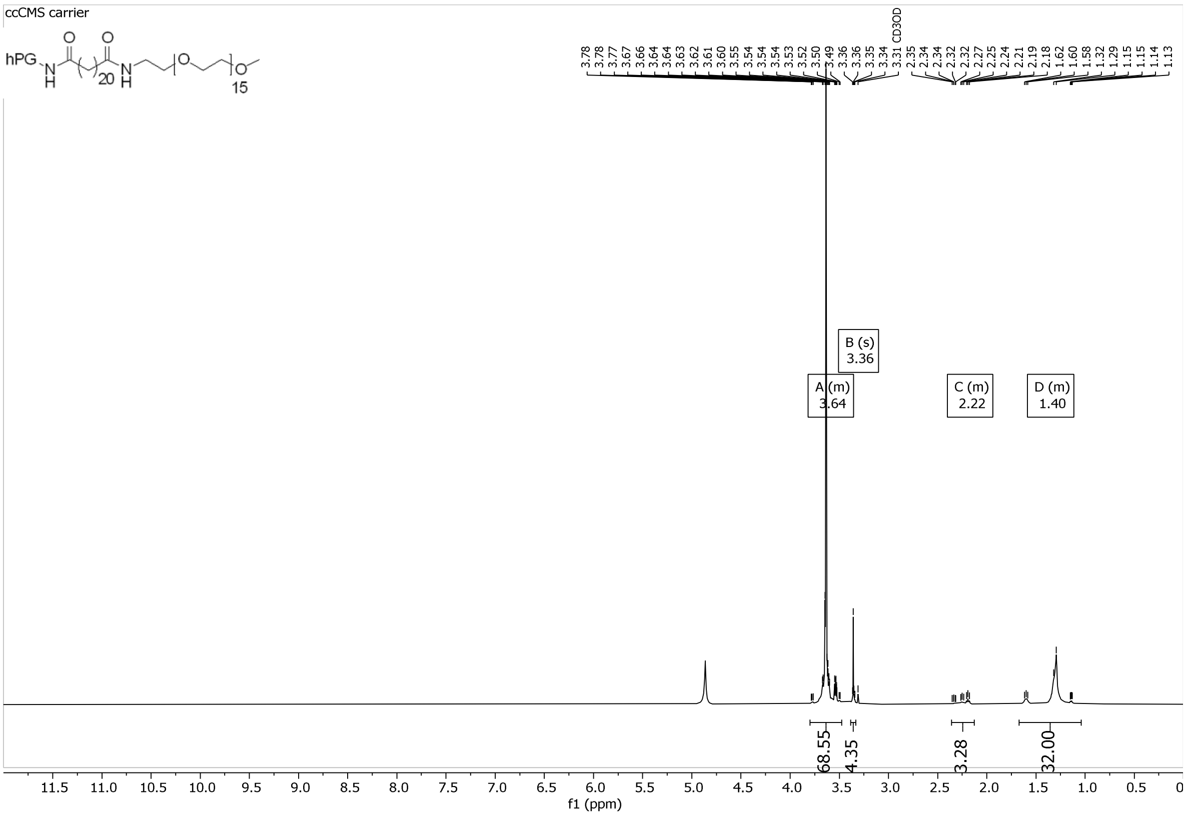

Figure S13. ^{1}H NMR of ccCMS **1b**.

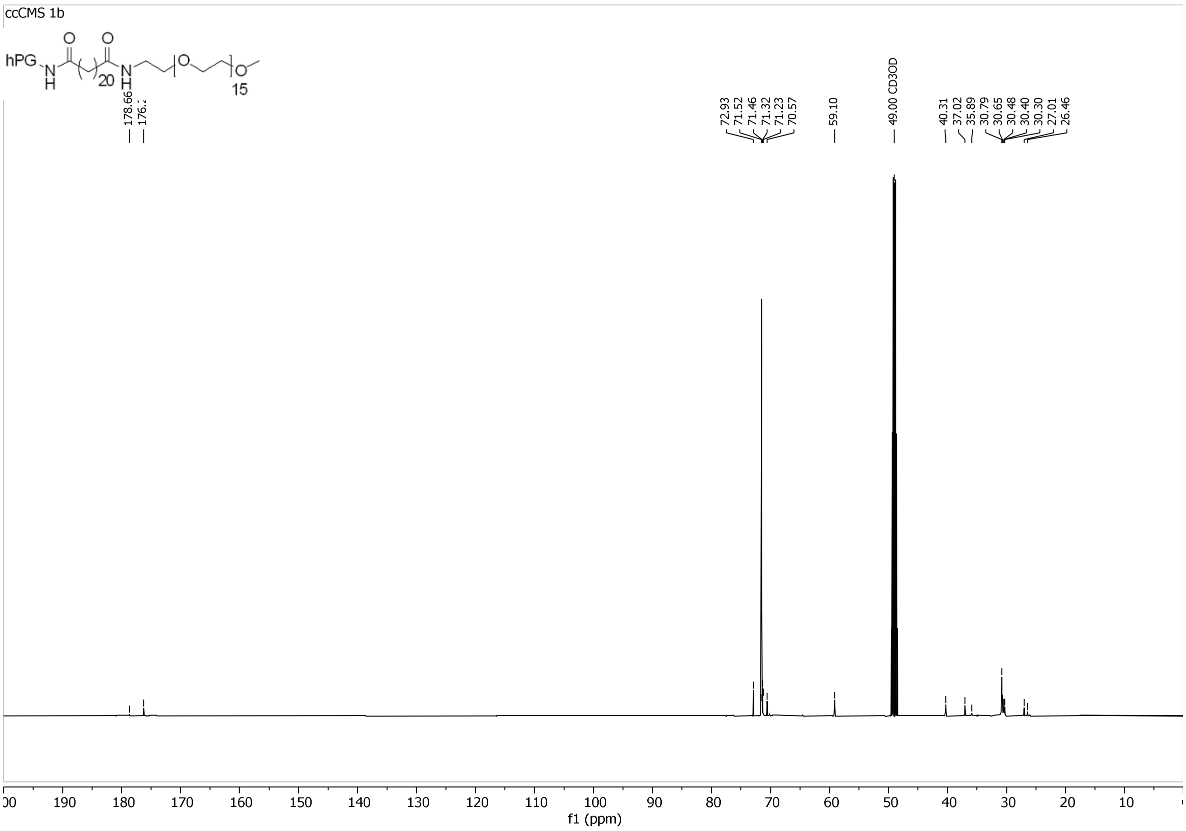

Figure S14. ^{13}C NMR of ccCMS **1b**.

2. Molar mass calculation via ^{1}H NMR

The calculation of the molar mass by ^{1}H NMR spectroscopy is based on the calculation of the degree of functionalization DF, reflecting the fraction of double shell conjugation to the hPG scaffold. Comparison of the polymeric hPG and PEG methylene and methine signals (around 3.7 ppm) to the respective alkyl backbone peaks of the double shell at around 1.2 ppm leads to the DF value [15].

- $\Sigma_{3.7}$ = Sum of hPG and PEG CH_2 and CH, in which hPG contributes five protons and PEG (mPEG=750 $g mol^{-1}$, two protons adjacent to amide shifted to higher ppm and thus subtracted)
- Aliphatic CH_2 set to 32

$$\sigma_{3.7} = 5 + 63 \times \mathrm{DF} - 2 \times \mathrm{DF} \quad (1)$$

$$\sigma_{3.7} = 5 + 61 \times \mathrm{DF} \quad (2)$$

$$\sigma_{1.2} = 32 \times \mathrm{DF} \quad (3)$$

$$\Sigma_{3.7} = \frac{\sigma_{3.7}}{\sigma_{1.2}} \times 32 = \frac{5 + 61 \times \mathrm{DF}}{\mathrm{DF}} \quad (4)$$

$$\Sigma_{3.7} = \frac{5}{\mathrm{DF}} + 61 \quad (5)$$

$$\mathrm{DF} = \frac{5}{\Sigma_{3.7} - 61} \quad (6)$$

$$DF_{rsCMS} = \frac{5}{66.6 - 61} = 89\ \% \tag{7}$$

$$DF_{ccCMS} = \frac{5}{68.5 - 61} = 67\ \% \tag{8}$$

3. Calibration curves for the determination of dexamethasone and rapamycin through HPLC

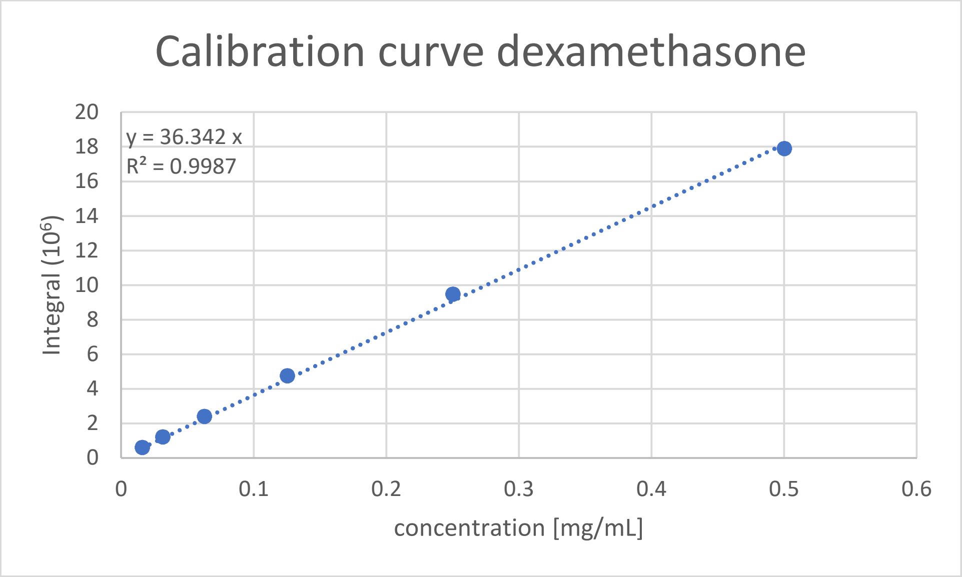

Figure S15. Calibration curve of dexamethasone.

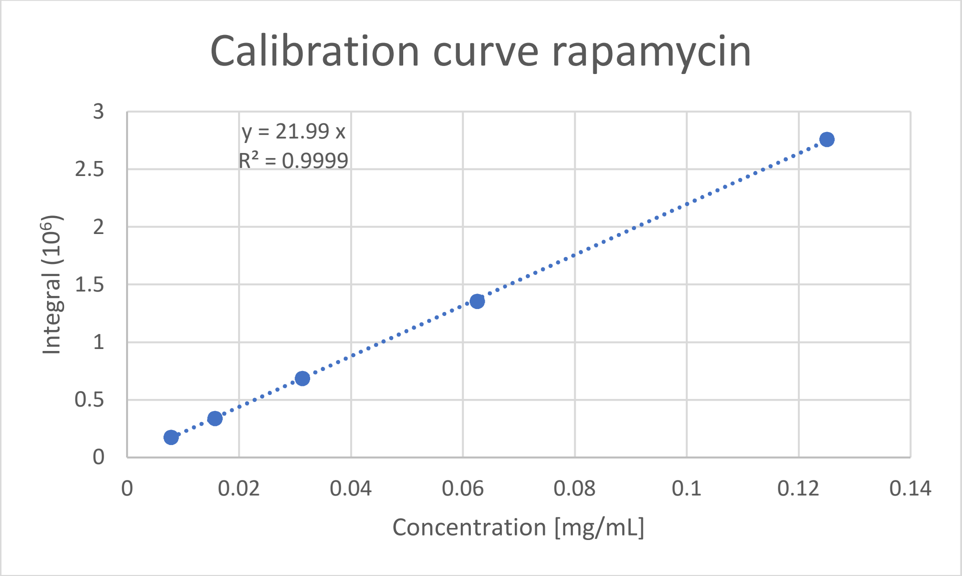

Figure S16. Calibration curve of rapamycin.

4. Cyclic voltammetric measurements

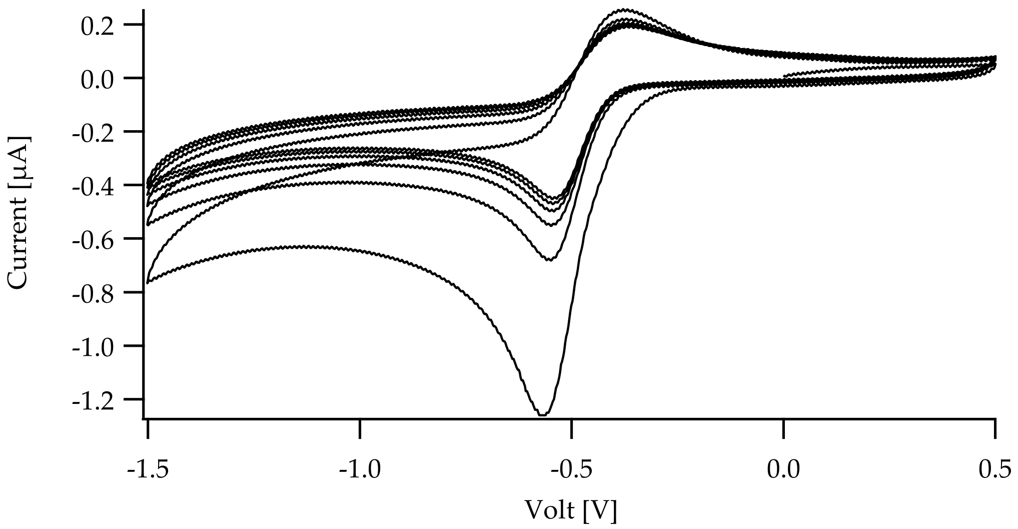

Figure S17. Cyclic voltammetric measurements of disulfide **6a** in dry DMSO at a scan rate of 100 mV/s, six rounds.

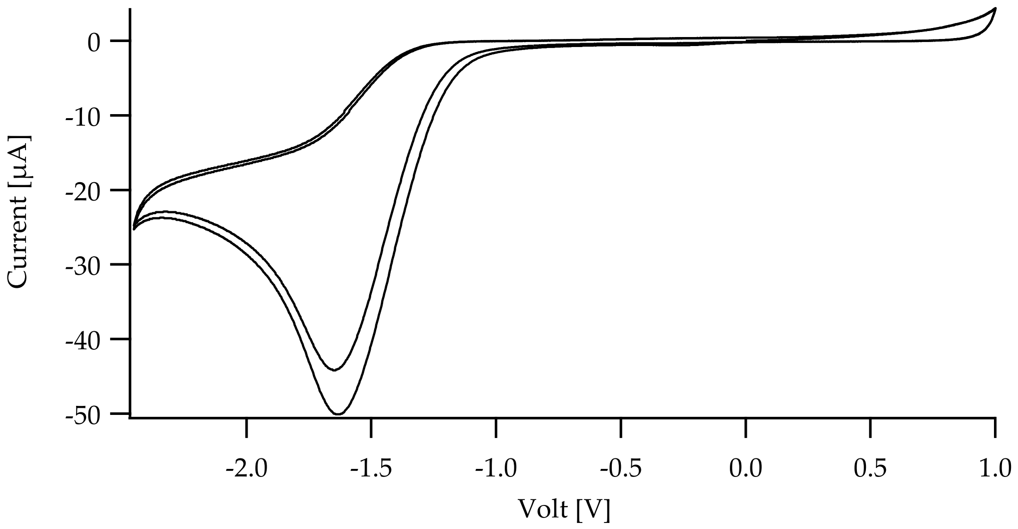

Figure S18. Cyclic voltammetric measurements of TCEP in dry DMSO at a scan rate of 100 mV/s, two rounds.

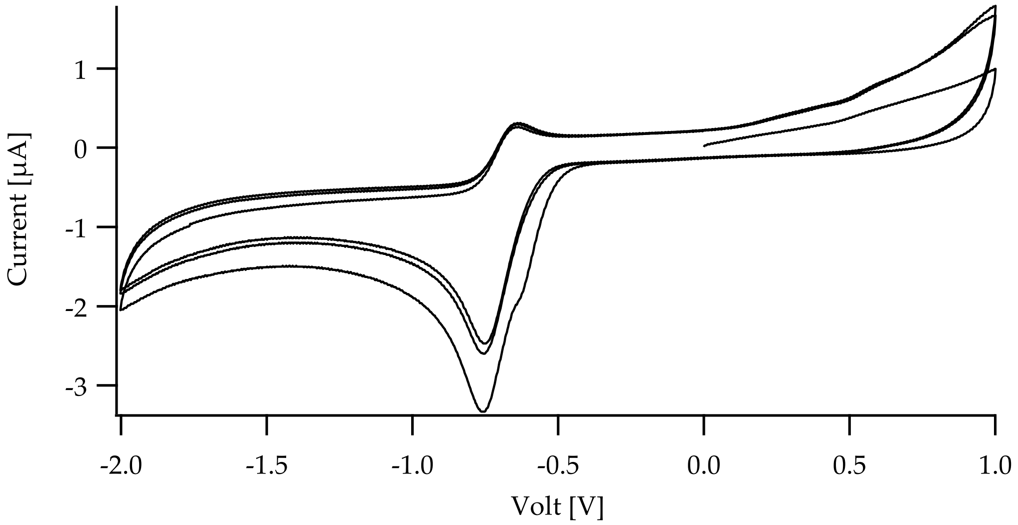

Figure S19. Cyclic voltammetric measurements of GSH in dry DMSO at a scan rate of 100 mV/s, three rounds.

5. *In vitro* proof-of-concept: triggered release

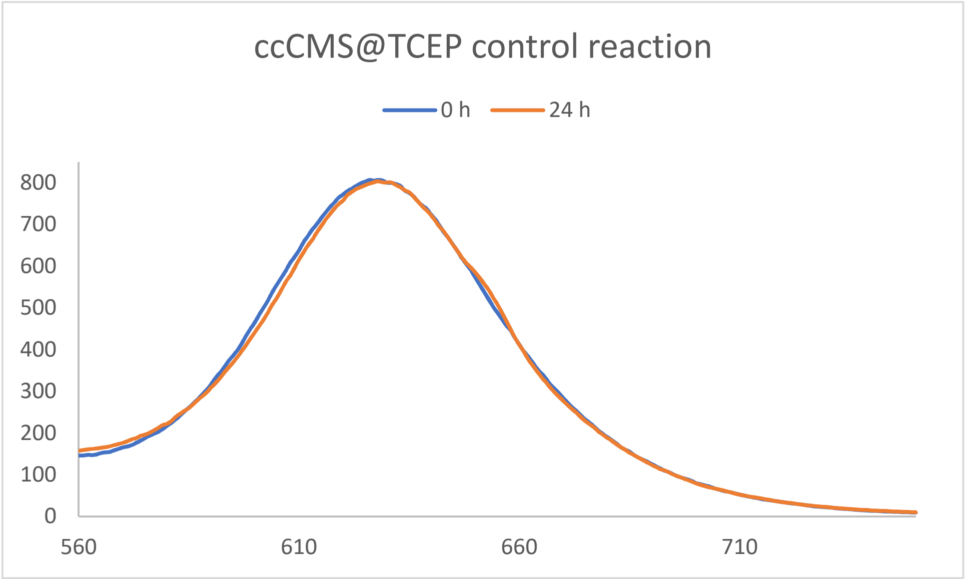

Figure S20. Fluorescence measurement of NR encapsulated ccCMS incubated with TCEP before and after 24 h.

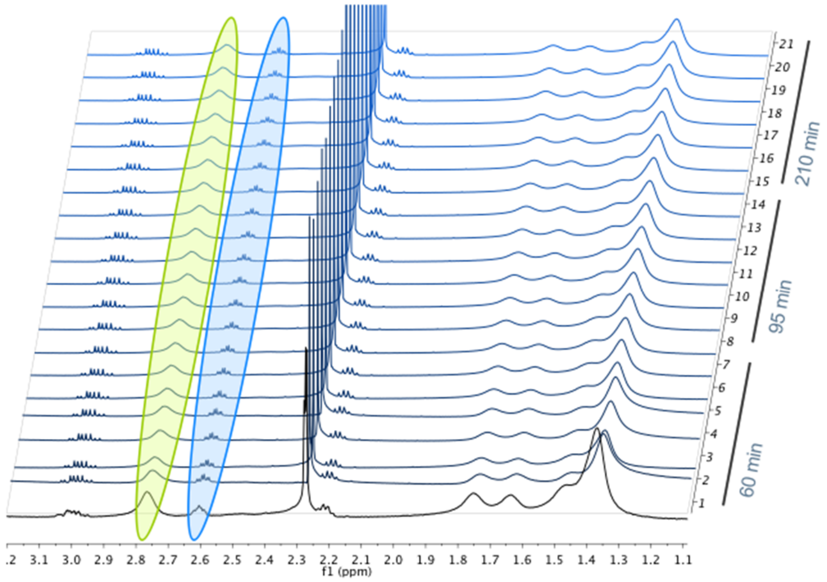

Figure S21. Stacked ^{1}H NMR spectra of interval measurement, 80 scans per measurement; incubation with 10 mM GSH solution in PBS pH 7.4 at 37 °C; ratio of 2.8 ppm (CH_2-SS-CH_2) vs 2.6 ppm (CH_2-SH).
